# High-affinity multivalent wheat germ agglutinin ligands by one-pot click reaction

**DOI:** 10.3762/bjoc.8.91

**Published:** 2012-06-01

**Authors:** Henning S G Beckmann, Heiko M Möller, Valentin Wittmann

**Affiliations:** 1 Fachbereich Chemie and Konstanz Research School Chemical Biology (KoRS-CB), Universität Konstanz, 78457 Konstanz, Germany

**Keywords:** carbohydrates, click chemistry, cluster effect, lectins, multivalency

## Abstract

A series of six mono-, di-, and trivalent *N*,*N*’-diacetylchitobiose derivatives was conveniently prepared by employing a one-pot procedure for Cu(II)-catalyzed diazo transfer and Cu(I)-catalyzed azide–alkyne cycloaddition (CuAAC) starting from commercially available amines. These glycoclusters were probed for their binding potencies to the plant lectin wheat germ agglutinin (WGA) from *Triticum vulgaris* by an enzyme-linked lectin assay (ELLA) employing covalently immobilized *N*-acetylglucosamine (GlcNAc) as a reference ligand. IC_50_ values were in the low micromolar/high nanomolar range, depending on the linker between the two disaccharides. Binding enhancements β up to 1000 for the divalent ligands and 2800 for a trivalent WGA ligand, compared to *N*,*N*’-diacetylchitobiose as the corresponding monovalent ligand, were observed. Molecular modeling studies, in which the chitobiose moieties were fitted into crystallographically determined binding sites of WGA, correlate the binding enhancements of the multivalent ligands with their ability to bind to the protein in a chelating mode. The best WGA ligand is a trivalent cluster with an IC_50_ value of 220 nM. Calculated per mol of contained chitobiose, this is the best WGA ligand known so far.

## Introduction

The recognition of carbohydrate structures by carbohydrate binding proteins (lectins) plays a fundamental role in numerous intra- and intercellular events during development, inflammation, immune response, cancer metastasis, and pathogen–host interactions [1–2]. Inhibition of such interactions by high-affinity ligands is of high medicinal interest for the treatment of many human diseases. However, carbohydrate–protein interactions are often characterized by low binding affinities. A possible solution to compensate for these weak individual receptor–ligand interactions is the multivalent presentation of sugar epitopes on suitable scaffolds. This principle is not only used in nature but is also a valid strategy for the construction of artificial lectin ligands [3–13]. Prime examples are the recently described ligands for the Shiga-like [14–15] and cholera toxins [16–17] both belonging to the AB_5_ family of bacterial toxins.

The frequent observation that the binding affinity of a multivalent ligand increases exponentially with the number of binding sites has been termed the glycoside cluster effect [18–19]. Due to the exponential increase of binding affinities, the cluster effect often leads to the amplification of the binding selectivity. This was experimentally demonstrated, for example, by Mortell et al. while investigating glycopolymer ligands of concanavalin A (Con A) [20]. Whereas two diastereomeric (monovalent) C-glycosidic Con A ligands displayed only a small difference in the free energies of binding to Con A, a sizable difference was measured between the corresponding multivalent C-glycosides (calculated per monovalent ligand within the glycopolymer). Such effects can be analyzed in the context of the chelate effect [21], and a number of theoretical models to treat multivalent receptor–ligand interactions have been developed [22–27]. A simple conclusion following from these analyses is that multimerization of monovalent ligands with enhanced binding affinity can lead to multivalent ligands with disproportionally enhanced avidity. A prerequisite for an effective multivalency effect, however, is that the linking spacer between the individual epitopes has the correct geometry to allow a simultaneous multipoint association, i.e., a chelating binding mode.

Wheat germ agglutinin (WGA), besides other plant lectins such as Con A, has been intensively employed as a model lectin to study the influence of the structure of multivalent ligands on the binding affinity. WGA ligands of defined structure containing two to twelve GlcNAc residues obtained either by individual synthesis [28–36] or from screening of combinatorial libraries [37–38] have been reported. WGA is a 36 kDa plant lectin composed of two identical glycine- and cysteine-rich subunits [39] and is enriched in the seeds of *Triticum vulgaris*. It is specific for terminal *N*-acetylneuraminic acid and *N*-acetylglucosamine (GlcNAc) and has been shown to inhibit fungal growth through interaction with fungal cell-wall components [40–42] and to agglutinate transformed cells in vitro [43–44].

Recently, we determined the structural basis of multivalent binding to WGA by X-ray crystallography [36] and EPR spectroscopy [45]. Crystal structure analysis of a complex of WGA and four molecules of a divalent ligand containing two GlcNAc residues showed that each ligand bridged adjacent binding sites with a distance of approx. 13–14 Å between the anomeric oxygen atoms of the GlcNAc residues. This structure confirmed for the first time that all eight sugar binding sites of the WGA dimer [46] are simultaneously functional, and provides the basis for the design of new multivalent ligands with improved binding affinity.

Besides GlcNAc, WGA also binds to chitooligosaccharides with even higher affinity. The association constant for the WGA–*N*,*N*’-diacetylchitobiose interaction, for example, has been determined to be *K* ≈ 5 × 10^3^ to 2 × 10^4^ M^–1^. The corresponding value for binding to GlcNAc is *K* ≈ 2 × 10^2^ to 1.3 × 10^3^ M^−1^ [47]. This prompted us to design a series of multivalent WGA ligands containing two or three *N*,*N*’-diacetylchitobiose moieties. To connect the chitobiose moieties we chose several linkers of varying length and flexibility, which were, nevertheless, all expected to allow simultaneous binding to adjacent binding sites in a chelating fashion, thus, leading to especially effective ligands. In this report, we describe the preparation of such a series of multivalent WGA ligands by a one-pot procedure for diazo transfer and azide–alkyne cycloaddition [48] starting from commercially available di- and triamines and the propargyl glycoside of *N*,*N*’-diacetylchitobiose. Binding potencies were determined by an enzyme-linked lectin assay (ELLA), resulting in IC_50_ values in the low-micromolar/high-nanomolar range. A trivalent ligand has a remarkable IC_50_ value of 220 nM. Molecular dynamics calculations based on published X-ray crystal structures of WGA-ligand complexes provide an explanation for the observed binding affinities.

## Results and Discussion

### Synthesis of glycoclusters

**Figure 1:**
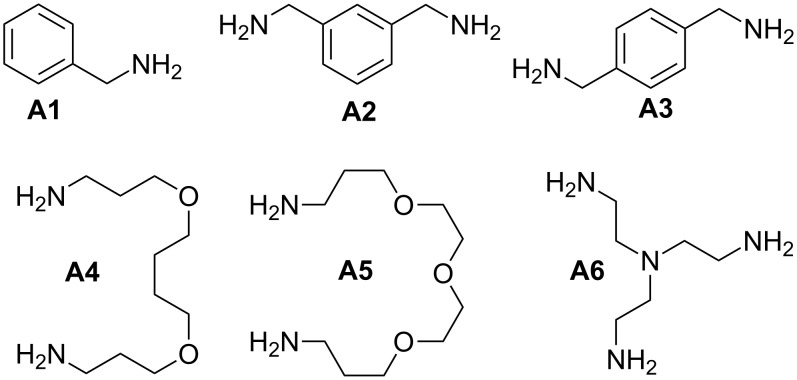
Amines used for the synthesis of glycoclusters.

The Cu(I)-catalyzed [49–50] Huisgen [3 + 2] cycloaddition [51] of azides and alkynes (CuAAC) is a frequently used method for the covalent attachment of carbohydrate epitopes to azide- or alkyne-presenting scaffolds [52–54]. Recently, we reported a convenient one-pot procedure for diazo transfer and azide–alkyne cycloaddition [48] giving access to multivalent triazole-linked structures starting from amines. For the synthesis of triazole-linked glycoclusters, commercially available amines **A1**–**A6** (Figure 1) comprising different spacer geometries were selected. These amines were employed in the sequential one-pot procedure [48] for diazo transfer and CuAAC (Table 1). First, the Cu(II)-catalyzed diazo transfer was performed at ambient temperature until complete conversion of the amines to azides. Then, CuAAC was provoked without any workup procedure by the addition of tris(benzyltriazolylmethyl)amine [55] (TBTA), sodium ascorbate, and the propargyl glycoside **1** [56] of *N*,*N*’-diacetylchitobiose and heating of the mixture to 80 °C by microwave irradiation, until TLC showed complete consumption of the intermediate azides (see Supporting Information File 1 for full experimental data).

**Table 1:** Synthesis of glycoclusters **B1**–**B6** using the one-pot procedure for diazo transfer and azide-alkyne cycloaddition.

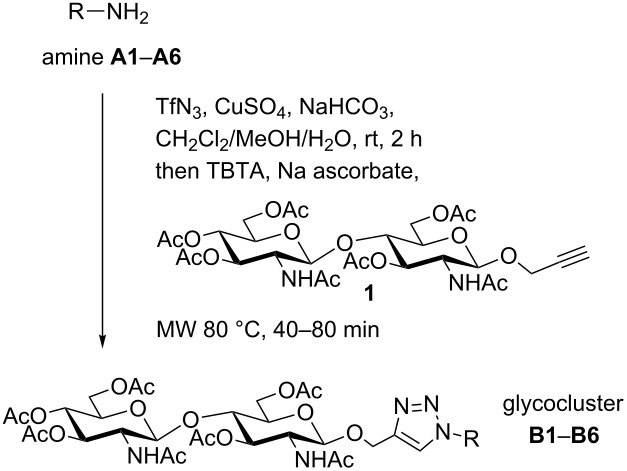		

Amine	Product	Yield (%)

**A1**	**B1**	45
**A2**	**B2**	30
**A3**	**B3**	37
**A4**	**B4**	28
**A5**	**B5**	48^a^
**A6**	**B6**	6

^a^In this case the intermediate diazide **2** was isolated (cf. Scheme 1).

**Scheme 1:**
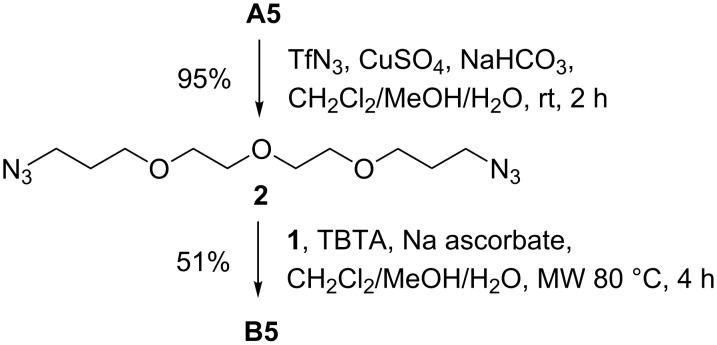
Synthesis of glycocluster **B5** with isolation of the intermediate diazide **2**.

**Scheme 2:**
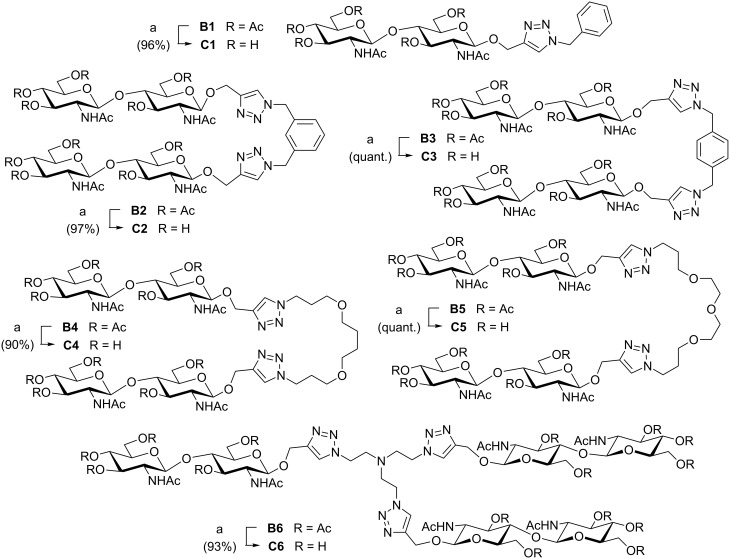
Deacetylation of glycoconjugates **B1**–**B6**. (a) NaOMe, MeOH.

According to TLC all reactions (except for **B6**) proceeded with complete conversion of the amines to the desired glycoconjugates. However, some loss of material during purification of the acetylated chitobiose derivatives by flash chromatography on silica gel led to the moderate yields indicated in Table 1. Monovalent compounds resulting from partial reactions of the diamines were not observed. To exclude that the observed yields are a result of the one-pot procedure, the preparation of divalent **B5** was carried out in two separate steps (Scheme 1). Diazo transfer with **A5** gave diazide **2** in a yield of 95%. Subsequent CuAAC of isolated **2** with alkyne **1** delivered **B5** after flash chromatography in 51% yield with no observed side products. Severe loss of about 50% of the material during flash chromatography was also experienced when a pure sample of disaccharide **1** was eluted from a silica gel column for a second time. In comparison, the use of the less polar propargyl β-D-glucoside instead of **1** in the one-pot procedure with amine **A4** led to the corresponding divalent glycocluster in a yield of 86% [48]. Finally, *O*-deacetylation of the glycoclusters **B1**–**B6** under Zemplén conditions resulted in WGA ligands **C1**–**C6** (Scheme 2).

**Scheme 3:**
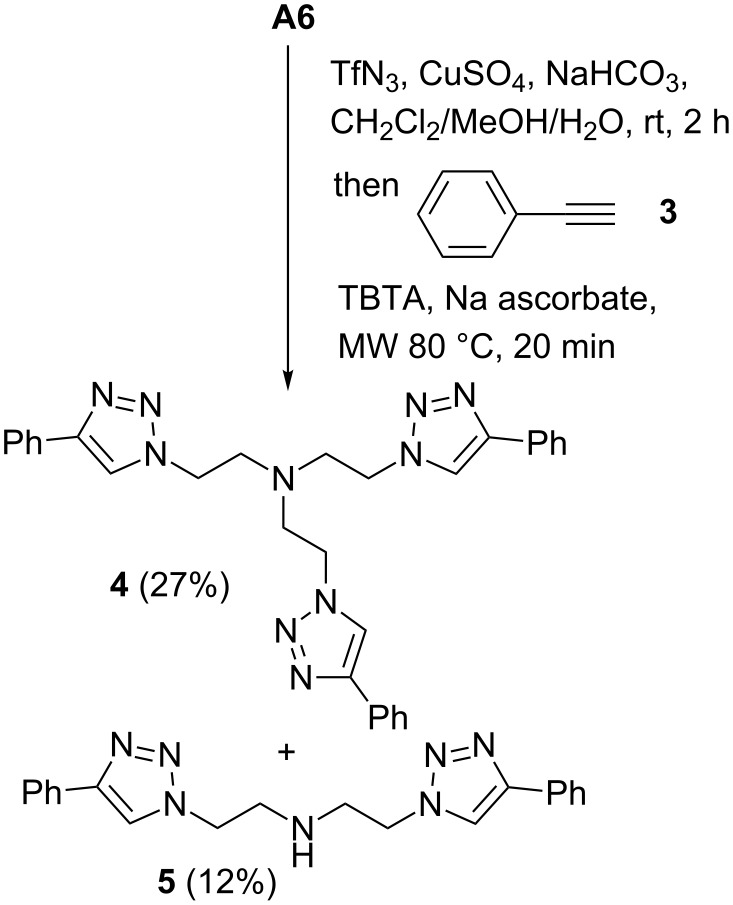
Formation of side-product **5** during the synthesis of **4**.

During the synthesis of trivalent compound **B6** the formation of a side product with a *R*_f_ similar to that of **B6** was observed. This is remarkable because all other reactions proceeded without the formation of side-products. The mass of this side product (*m*/*z* [M + H]^+^ = 1500.7) corresponds to a divalent compound in which one arm of the tertiary amine is missing. Since this side product could not be isolated in pure form, we investigated the reaction of **A6** with phenylacetylene (**3**, Scheme 3). Also with this alkyne two main products were obtained which were difficult to separate by chromatography. Apart from the expected tris(triazole) **4**, the secondary amine **5** was isolated and characterized. This structure corresponds to the assumed side-product obtained during the synthesis of **B6**. A contamination of the starting material **A6** with secondary amine di(2-aminoethyl)amine was excluded. ESI–MS measurements indicated that side-product formation may already take place during the diazo transfer reaction of **A6** because the mass of the corresponding intermediate di(2-azidoethyl)amine (*m*/*z* [M + H]^+^ = 156.2) was found. The mechanism of this side product formation is not clear. We assume that the mechanism is due to the special structure of **A6** because comparable side-product formation was not observed with any other amine used.

### Determination of binding potencies by ELLA

**Figure 2:**
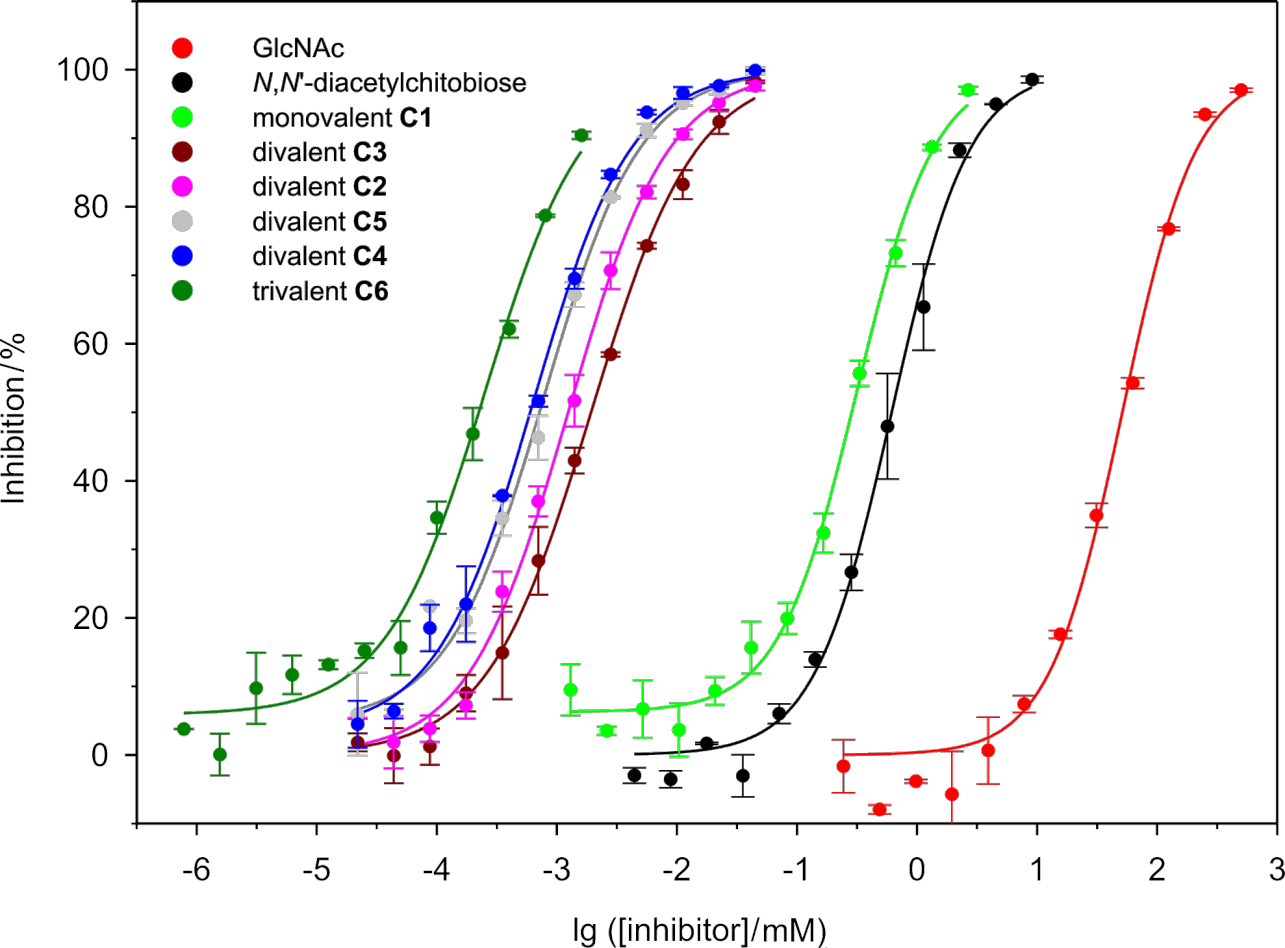
Dose-response curves for the inhibition of binding of HRP-labeled WGA to covalently immobilized GlcNAc by synthetic ligands **C1**–**C6**.

Binding potencies of compounds **C1**–**C6**, GlcNAc, and *N*,*N*’-diacetylchitobiose were determined by an ELLA employing covalently immobilized GlcNAc as a reference ligand, as described recently [34]. GlcNAc-coated microtiter plates were incubated with mixtures of horseradish-peroxidase-labeled WGA (HRP-WGA) and synthetic WGA ligands in varying concentrations. After incubation, the plates were washed and the remaining HRP-WGA bound to the microtiter plates was quantified by a HRP-catalyzed color reaction. Dose-response curves for inhibition of the binding of HRP–WGA to immobilized GlcNAc are shown in Figure 2. From these curves the concentrations at which the binding of HRP–WGA to GlcNAc is reduced by 50% (IC_50_ values) were determined as a measure of the potency of the synthesized inhibitors (Table 2). Also shown in Table 2 are the relative inhibitory potencies (β values) referenced to *N*,*N*’-diacetylchitobiose (β = 1).

**Table 2:** Absolute and relative IC_50_ values of synthetic ligands **C1**–**C6** for inhibition of the binding of HRP-labeled WGA to covalently immobilized GlcNAc from dose-response curves shown in Figure 2.

Compound	IC_50_ (µM)	β

GlcNAc	51000	1/82
*N*,*N*'-diacetylchitobiose	620	1
monovalent **C1**	290	2.1
divalent **C2**	1.3	480
divalent **C3**	1.9	330
divalent **C4**	0.60	1000
divalent **C5**	0.72	860
trivalent **C6**	0.22	2800

With an 82 times lower IC_50_ value, *N*,*N*’-diacetylchitobiose is a much better inhibitor than GlcNAc, which is in accordance with the association constants determined by solution binding assays [47]. The benzyl triazolyl appendage of **C1** further enhances binding by a factor of two, probably due to additional weak hydrophobic interactions. However, introducing an additional *N*,*N*’-diacetylchitobiose epitope leads to dramatically increased affinities. The divalent chitobiose derivatives **C2**–**C5** have IC_50_ values in the low-micromolar/high-nanomolar range, with some variation due to different spacer properties. Their inhibitory potencies relative to *N*,*N*'-diacetylchitobiose are 330–1000. It is interesting to note the differences induced by differing linker geometries. Here, not the most hydrophobic linkers show strongest binding but the ones that apparently promote multivalent binding most efficiently. Whereas the flexible linkers of **C4** and **C5** lead to β values of 1000 and 860, respectively, the ligands **C2** and **C3** with the less flexible aromatic linkers have significantly lower binding potencies (β values: 480 and 330). This observation points to the possibility that the aromatic linkers cannot adopt a strain-free conformation if the ligand binds in a chelating mode to WGA. The best divalent WGA ligand is **C4** (IC_50_ 0.6 µM), which binds 1000 times stronger to WGA than *N*,*N*’-diacetylchitobiose (500 times per chitobiose residue).

As expected, clustering of carbohydrate epitopes with higher WGA binding affinity not only leads to multivalent ligands with higher absolute affinity but also to a higher binding enhancement β relative to the respective monovalent compound. Earlier, we reported WGA binding affinities of β-*O*-glycosidic divalent GlcNAc derivatives with linker lengths comparable to those of **C2**–**C5** [34]. These GlcNAc derivatives displayed β values of 80–260 relative to GlcNAc, which are much lower than the β values (330–1000, relative to *N*,*N*'-diacetylchitobiose) determined for **C2**–**C5** (Table 2). The relative potency of **C4** of 500 per chitobiose residue is even higher than that of tetra- to octavalent GlcNAc clusters [28,31–34,37]. We are aware of only one example of a divalent GlcNAc derivative with an exceptional β value of 2350 [36]. In this case, however, the GlcNAc moieties are α-*O*-glycosidically linked.

For trivalent cluster **C6** an IC_50_ value of 220 nM (660 nM per contained chitobiose) was determined, which is 2800-fold lower than that of *N*,*N*'-diacetylchitobiose or 230000-fold lower than the IC_50_ value of GlcNAc. This is one of the best WGA ligands known. Masaka et al. reported a tetravalent *N*,*N*’-diacetylchitobiose derivative with an IC_50_ value of 180 nM (720 nM per contained chitobiose) determined by a hemagglutination inhibition assay [57]. Calculating the IC_50_ value per contained chitobiose, trivalent **C6** is the better ligand. However, since such numbers are strongly dependent on the employed assay [58–59], they cannot be readily compared. Interestingly, the ligand reported by Masaka et al. led to precipitation of WGA. In this respect it is worth mentioning that we never observed precipitate formation during incubation of WGA with our synthetic ligands. This suggests that in our case intermolecular multivalency (cross-linking) plays a negligible role and that the main mechanism of affinity enhancement is chelating binding to the same WGA dimer.

The 2.8-fold increased inhibition potency of **C6** over the best divalent ligand **C4** indicates that **C6** can reach only two WGA binding sites simultaneously due to its geometrical properties, which is fully in accordance with the structural investigations described below. Otherwise, a significantly stronger binding enhancement would have been expected comparable to the several-hundred-fold increase observed when moving from mono- to divalent ligand structures.

### Molecular modeling

To provide a structure-based rationalization for the determined binding potencies of **C2**–**C6**, we performed molecular modeling studies. Combining information from the crystal structures of WGA3 in complex with *N*,*N*’-diacetylchitobiose (PDB ID: 1K7U) [60] and WGA3 binding to a divalent ligand presenting two GlcNAc residues (PDB ID: 2X52) [36], two *N*,*N*’-diacetylchitobiose residues were placed in a pair of adjacent primary binding sites of WGA and connected by the respective linker (see Supporting Information File 1 for details). Subsequently, the linkers were energy minimized with the chitobiose moieties fixed in their ideal positions. The models containing the various linker structures were further energy minimized with the terminal GlcNAc residues kept in their optimal positions.

**Figure 3:**
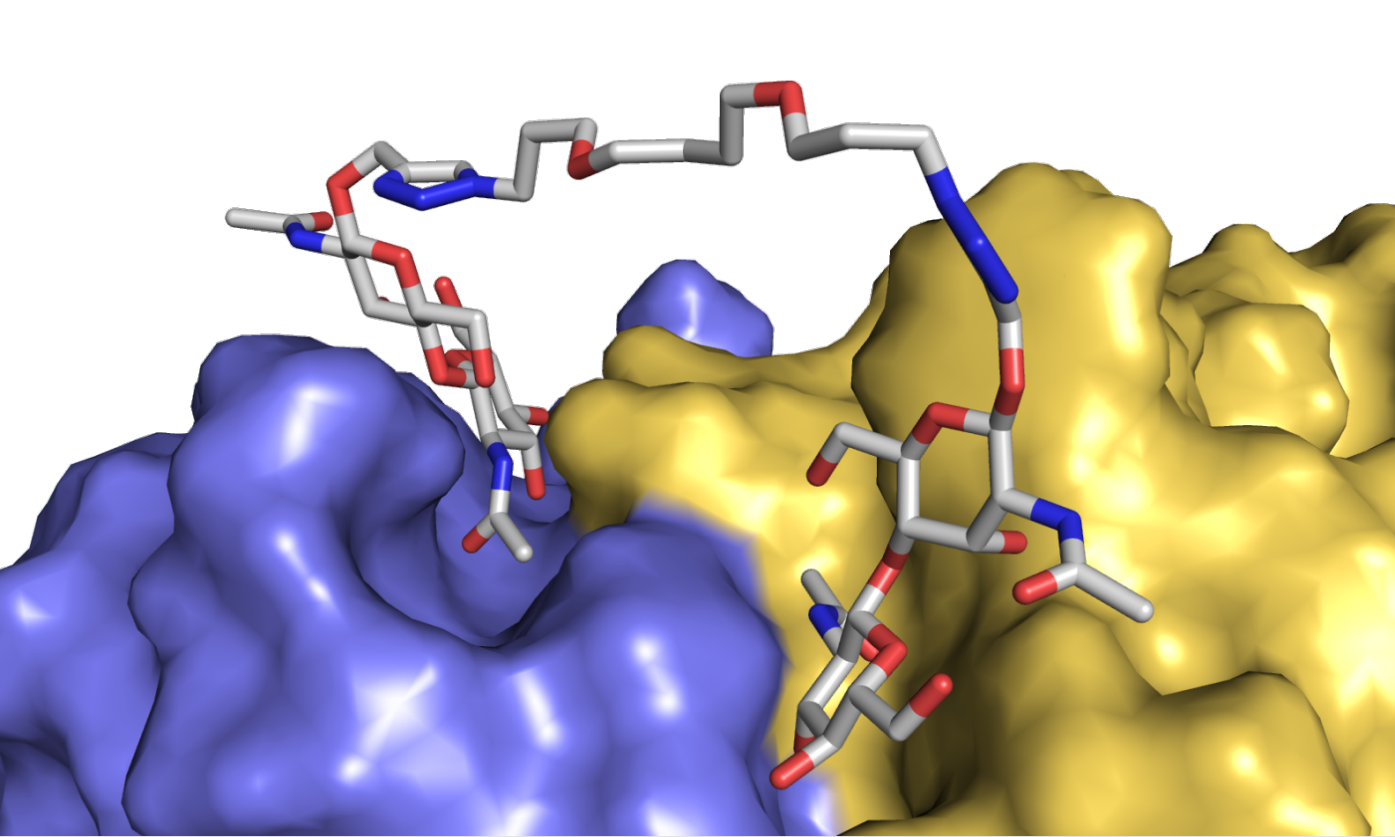
Molecular model of divalent ligand **C4** with its two chitobiose moieties occupying two adjacent binding sites of WGA. The linker is conformationally largely unrestricted and can adopt several low-energy conformations. WGA chain 1 is colored yellow, chain 2 is blue.

Our molecular modeling studies revealed that the *para*-disubstituted aromatic linker of **C3** cannot adopt a low-energy conformation if the chitobiose residues are kept in their ideal positions in the binding sites of WGA, resulting in significant ring strain of the triazole moieties as well as the central phenyl ring. This ring strain can be reduced by slightly pulling the GlcNAc residues directly attached to the linker out of the binding site, but at the expense of a less efficient multivalent binding of the two chitobiose entities. The chitobiose groups of the divalent ligand **C4**, on the other hand, can maintain their ideal positions easily with the linker adopting an all-staggered low-energy conformation (Figure 3). Ligand **C2** allows for the positioning of its chitobiose moieties ideally in both binding sites when adopting a fully extended conformation. Ligand **C2**, and even more so ligand **C3**, possess very limited conformational freedom when adopting a chelating binding mode. Conformational changes within the linkers of **C2** and **C3** lead to forces that pull one or the other GlcNAc/chitobiose out of its binding site. This situation may increase the entropic costs of chelating binding for these two ligands providing a further explanation for their significantly lower binding affinity.

With its linker length and flexibility, trivalent ligand **C6** is in an intermediate position between the relatively short-bridged and rigid ligands **C2** and **C3** and the more flexibly connected ligands **C4** and **C5**. With six chemical bonds between the triazole groups, **C6** can present its *N*,*N*’-diacetylchitobiose residues in rather similar distances as **C2** or **C3** having the same linker length. However, **C6** has two more rotatable bonds between pairs of chitobiose moieties leading to significantly increased conformational freedom. This is expected to facilitate binding in a chelating fashion at relatively low entropic costs. It is important to note that the third chitobiose unit of **C6** cannot reach a third carbohydrate binding site of the WGA dimer. The closest distance to another binding site is approximately 24 Å (measured between the anomeric oxygens of the inner GlcNAc residues). The 2.8-fold increased potency relative to ligand **C4** could possibly originate from the facilitated rebinding of monovalently bound trivalent ligand **C6** due to the two-fold-higher local concentration of chitobiose compared to the divalent ligands.

## Conclusion

In summary, we have presented a series of *O*-glycosidically linked *N*,*N*’-diacetylchitobiose clusters that were conveniently obtained from propargyl glycoside **1** and readily available amine scaffolds by a one-pot procedure for diazo transfer and azide–alkyne cycloaddition. Binding potencies were determined by an ELLA. Divalent ligands were found to have IC_50_ values in the low-micromolar/high-nanomolar range depending on the linker between the two disaccharides. The observed binding enhancements over the monovalent ligand are significantly higher than those of comparable β-linked GlcNAc clusters. The different binding enhancements can be rationalized by molecular modeling studies that correlate the different linker geometries with their propensities to support chelating binding. The best WGA ligand is trivalent cluster **C6** with a remarkable IC_50_ value of 220 nM. Calculated per mol of contained chitobiose, this is the best WGA ligand published so far.

## Supporting Information

File 1 Experimental procedures and analytical data for all new compounds.
