# Supplementary material for: High-affinity multivalent wheat germ agglutinin ligands by one-pot click reaction
Source: Beilstein J Org Chem. 2012 Jun 1;8:819–26. doi: 10.3762/bjoc.8.91 (PMC3388869; doi:10.3762/bjoc.8.91)
Supplement: File 1 — Experimental procedures and analytical data for all new compounds. [file Beilstein_J_Org_Chem-08-819-s001.pdf]

Supporting Information

for

High-affinity multivalent wheat germ agglutinin ligands by one-pot click reaction

Henning S. G. Beckmann, Heiko M. Möller and Valentin Wittmann*

Address: Fachbereich Chemie and Konstanz Research School Chemical Biology (KoRS-CB), Universität Konstanz, 78457 Konstanz, Germany

Email: Valentin Wittmann* - mail@valentin-wittmann.de

* Corresponding author

Experimental procedures and analytical data for all new compounds

Contents

General methods S2

Synthesis of WGA ligands S3

Determination of binding potencies of WGA ligands S12

Molecular modeling S13

References S13

HPLC chromatograms S15

^{1}H NMR spectra S23

Experimental

General methods

Propargyl 2-acetamido-3,6-di-*O*-acetyl-2-deoxy-4-*O*-(2-acetamido-3,4,6-tri-*O*-acetyl-2-deoxy-β-D-glucopyranosyl)-β-D-glucopyranoside (**1**) was prepared as described before [1]. Syntheses under microwave irradiation were performed in sealed tubes by using a Biotage AB SmithSynthesizer. TLC was performed on Merck Silica Gel 60 F_{254} aluminum sheets. Detection by UV light was used when applicable. Reagents used for developing plates include cerium reagent (5 g molybdatophosphoric acid, 2.5 g ceric sulfate tetrahydrate, 25 mL sulfuric acid and 225 mL water), ethanolic ninhydrin (3% w/v), and ethanolic sulfuric acid (15% v/v). Flash column chromatography (FC) was performed on Macherey-Nagel Silica Gel 60 (0.04–0.063 mm; 230–400 mesh ASTM). RP-HPLC was performed on a Shimadzu LC-20A prominence system using a Knauer Nucleosil 100-5 C-18 column (4 × 250 mm, flow 0.9 mL min^{-1}) and gradients of water with 0.1% TFA (eluent A) and increasing proportions of acetonitrile with 0.1% TFA (eluent B). ^{1}H and ^{13}C NMR spectra were recorded at room temperature on Bruker Avance DRX 600 and Avance III 400 instruments. ^{1}H chemical shifts are given in ppm referenced to residual protic solvent ($CDCl_3$, δ_H = 7.26 ppm; DMSO-d_6, δ_H = 2.50 ppm; D_2O, δ_H = 4.67 ppm; CD_3OD, δ_H = 3.31 ppm). ^{13}C chemical shifts are given in ppm referenced to the solvent signal ($CDCl_3$, δ_C = 77.0 ppm; DMSO-d_6, δ_C = 39.4 ppm; methanol-d_4, δ_C = 49.1 ppm). Assignments of proton and carbon resonances were carried out with the aid of DQF-COSY and HSQC experiments. ESI-IT mass spectra were recorded on a Bruker Daltonics Esquire 3000 plus instrument. High-resolution ESI-TOF mass spectra were recorded on a Bruker Daltonics micrOTOF II instrument. High-resolution MALDI-FTICR mass spectra were recorded on a Bruker Daltonics Aplex II - FTICR instrument. Combustion elemental analyses were performed on an elementar vario EL analyzer.

Warning: In the case of reactions with azide sources in dichloromethane, formation of explosive diazidomethane has been reported [2-7]. Therefore, the triflyl azide solution in dichloromethane should always be prepared freshly and the reaction time of 2 h should not be exceeded. Special caution should also be exercised during the

workup procedure (especially during evaporation of the reaction mixture) due to excess triflyl azide and possibly formed copper azides, although we never observed any incident.

Synthesis of WGA ligands

General procedure 1 (GP 1): Synthesis of triazole-linked glycoclusters using a sequential one-pot process for diazo transfer and azide–alkyne cycloaddition

This one-pot process was carried out in a similar way as described previously [8]. Triflyl azide (TfN_3) was freshly prepared prior to each reaction [9, 10]. NaN_3 (6 equiv per substrate amine) was dissolved in a minimum volume of water (solubility of NaN_3 in water is approximately 0.4 g mL^{-1}). At 0 °C, an equal volume of dichloromethane was added and triflic anhydride (Tf_2O) (3 equiv) was added dropwise to the vigorously stirred solution. After stirring for 2 h at 0 °C, the aqueous phase was extracted once with the same volume of dichloromethane. The combined organic phases were washed with sat. aq. $NaHCO_3$ solution and used without further purification.

The substrate amine, $CuSO_4$ (2–8 mol %) and $NaHCO_3$ (1 equiv) were dissolved/ suspended in the same volume of water as the volume of the TfN_3 solution to be employed. The TfN_3 solution was added, followed by the addition of methanol until the solution became homogeneous. The reaction mixture was stirred at room temperature (ca. 2 h) until TLC showed complete conversion of the starting amine.

Then, propargyl glycoside **1** (1–1.2 equiv), TBTA [11] (5 mol %) and sodium ascorbate (0.05–1.1 equiv per substrate amine) were added and the reaction mixture was heated to 80 °C in the microwave oven until TLC showed complete conversion of the azide intermediate (40–80 min).

The reaction mixture was diluted with water, the organic solvents were removed under reduced pressure, and the remaining water was removed by lyophilization. (In order to remove copper salts, it is advisable to stir the solution with CupriSorb resin (Seachem Labaratories, Madison, GA) and filter it before lyophilization.) The crude product was purified by flash chromatography.

General Procedure 2 (GP 2): Deacetylation under Zemplén conditions

The acetylated glycocluster was dissolved in dry methanol. If necessary, dry dichloromethane (up to a ratio of 1:1) was added to obtain a clear solution. Then, sodium methanolate solution (0.17–0.7 equiv per *N*,*N*'-diacetylchitobiose unit) was added. If a precipitate was formed during reaction, it was dissolved by the addition of a small amount of water. If necessary, additional sodium methanolate solution was added to complete the reaction. After completion of the reaction, the mixture was neutralized with acidic ion-exchange resin (DOWEX 50 WX8) and filtered. The organic solvent was removed under reduced pressure and the residue was dissolved in water and the resulting solution was lyophilized.

4-[2-Acetamido-3,6-di-*O*-acetyl-2-deoxy-4-*O*-(2-acetamido-3,4,6-tri-*O*-acetyl-2-deoxy-β-D-glucopyranosyl)-β-D-glucopyranosyloxymethyl]-1-benzyl-1*H*-1,2,3-triazole (B1)

Benzylamine **A1** (18 mg, 0.16 mmol) and propargyl glycoside **1** (108 mg, 0.16 mmol) were reacted according to GP 1 using 4 mol % $CuSO_4$ and 0.91 equiv sodium ascorbate. After FC (ethyl acetate/MeOH 9:1) **B1** (59 mg, 45%) was isolated as a white powder. ^{1}H NMR (DMSO-d_6, 600 MHz) δ 8.14 (d, *J* = 9.0 Hz, 1H, NH'), 8.08 (s, 1H, triazole-5), 7.94 (d, *J* = 9.0 Hz, 1H, NH), 7.37–7.28 (m, 5H, Ph), 5.57 (s, 2H, N-CH_2), 5.11 (ψt, *J* = 9.9 Hz, 1H, H-3'), 4.94 (ψt, *J* = 9.6 Hz, 1H, H-3), 4.79 (ψt, *J* = 9.7 Hz, 1H, H-4'), 4.72–4.67 (m, 2H, H-1', CH_aH_b), 4.66 (d, *J* = 8.5 Hz, 1H, H-1), 4.56 (d, *J* = 12.3 Hz, 1H, CH_aH_b), 4.36–4.32 (m, 1H, H-6a), 4.25 (dd, *J* = 3.8, 12.3 Hz, 1H, H-6a'), 4.05 (dd, *J* = 6.1, 12.0 Hz, 1H, H-6b), 3.89–3.85 (m, 1H, H-6b'), 3.81–3.77 (m, 1H, H-5'), 3.72 (ψt, *J* = 9.4 Hz, 1H, H-4) 3.64–3.52 (m, 3H, H-5, H-2, H-2'), 2.04 (s, 3H, C(O)CH_3), 1.99 (s, 3H, C(O)CH_3), 1.93 (s, 3H, C(O)CH_3), 1.92 (s, 3H, C(O)CH_3), 1.88 (s, 3H, C(O)CH_3), 1.75 (s, 3H, C(O)CH_3), 1.65 (s, 3H, C(O)CH_3); ^{13}C NMR (DMSO-d_6, 150.9 MHz) δ 170.1, 170.0, 169.6, 169.4, 169.3, 169.2, 169.1 (each C=O), 143.5 (triazole-4), 135.9, 128.7, 128.1, 127.9 (each Ph), 124.2 (triazole-5), 100.2 (C-1'), 99.3 (C-1), 75.9 (C-4), 73.5 (C-3), 72.3 (C-3'), 71.9 (C-5), 70.4 (C-5'), 68.2 (C-4'), 62.3 (C-6), 61.7 (CH_2), 61.5 (C-6'), 53.7 (C-2'), 53.3 (C-2), 52.7 (N-CH_2), 22.6, 22.5, 20.7, 20.4, 20.4, 20.4, 20.3 (each CH_3); *m*/*z* (MALDI–FTICR) for $C_{36}H_{47}N_5O_{16}$: calcd. 828.2910 $[M + Na]^+$; found 828.2890.

α,α'-Bis{4-[2-acetamido-3,6-di-*O*-acetyl-2-deoxy-4-*O*-(2-acetamido-3,4,6-tri-*O*-acetyl-2-deoxy-β-D-glucopyranosyl)-β-D-glucopyranosyloxymethyl]-1*H*-1,2,3-triazole-1-yl}-*m*-xylylene (B2)

α,α'-Diamino-*m*-xylylene (**A2**, 12 mg, 0.088 mmol) and propargyl glycoside **1** (145 mg, 0.216 mmol) were reacted according to GP 1 using 16 mol % $CuSO_4$ and 2.2 equiv sodium ascorbate. After FC (chloroform/MeOH 8:1) **B2** (41 mg, 30%) was isolated as a white powder. ^{1}H NMR ($CDCl_3$, 600 MHz) δ 7.52 (s, 2H, triazole-5), 7.35–7.30 (m, 3H, H-Ar5, NH), 7.24–7.21 (m, 4H, H-Ar4, H-Ar6, NH'), 6.80–6.76 (m, 1H, H-Ar2), 5.48 (d, *J* = 15.1 Hz, 2H, N-C$H_a$$H_b$), 5.43 (d, *J* = 15.2 Hz, 2H, N-C$H_a$$H_b$), 5.12 (ψt, *J* = 9.7 Hz, 2H, H-3'), 5.10–5.05 (m, 2H, H-3), 4.96–4.92 (m, 2H, H-4'), 4.79 (d, *J* = 12.8 Hz, 2H, C$H_a$$H_b$), 4.68–4.63 (m, 2H, C$H_a$$H_b$), 4.55–4.49 (m, 4H, H-1, H-1'), 4.34–4.28 (m, 4H, H-6a, H-6a'), 4.06–4.01 (m, 2H, H-6b), 3.97–3.91 (m, 4H, H-2, H-6b'), 3.79–3.70 (m, 2H, H-2'), 3.69–3.64 (m, 2H, H-4), 3.63–3.59 (m, 2H, H-5'), 3.56–3.52 (m, 2H, H-5), 2.03 (s, 6H, C(O)CH_3), 2.00 (s, 6H, C(O)CH_3), 1.95–1.92 (m, 18 H, C(O)CH_3), 1.83 (s, 6H, C(O)CH_3), 1.73 (s, 6H, C(O)CH_3); ^{13}C NMR ($CDCl_3$, 150.9 MHz) δ 171.2, 171.1, 171.0, 170.7, 170.7, 170.6, 169.5 (each C=O), 144.4 (triazole-4), 135.9 (Ar-1, Ar-3), 129.6 (Ar-5), 127.9 (Ar-4, Ar-6), 126.2 (Ar-2), 123.5 (triazole-5), 100.8 (C-1'), 99.7 (C-1), 76.0 (C-4), 72.9 (C-3), 72.6 (C-5), 72.3 (C-3'), 71.4 (C-5'), 68.1 (C-4'), 62.4 (C-6), 61.9 (CH_2), 61.6 (C-6'), 54.3 (C-2'), 53.4 (N-CH_2), 53.3 (C-2), 22.6, 22.5, 20.7, 20.7, 20.5, 20.4, 20.4 (each CH_3); *m*/*z* (MALDI–FTICR) for $C_{66}H_{88}N_{10}O_{32}$: calcd. 1555.5458 $[M + Na]^+$; found 1555.5489.

α,α'-Bis{4-[2-acetamido-3,6-di-*O*-acetyl-2-deoxy-4-*O*-(2-acetamido-3,4,6-tri-*O*-acetyl-2-deoxy-β-D-glucopyranosyl)-β-D-glucopyranosyloxymethyl]-1*H*-1,2,3-triazole-1-yl}-*p*-xylylene (B3)

α,α'-Diamino-*p*-xylylene (**A3**, 12 mg, 0.088 mmol) and propargyl glycoside **1** (130 mg, 0.194 mmol) were reacted according to GP 1 using 4 mol % $CuSO_4$ and 0.1 equiv sodium ascorbate. After FC (chloroform/MeOH 8:1) **B3** (50 mg, 37%) was isolated as a white powder. ^{1}H NMR (DMSO-d_6, 600 MHz) δ 8.07–8.00 (m, 4H, NH', triazole-5), 7.87–7.81 (m, 2H, NH), 7.29 (s, 4H, H-Ar), 5.56 (s, 4H, N-CH_2), 5.12 (ψt, *J* = 10.0 Hz, 2H, H-3'), 4.94 (ψt, *J* = 9.6 Hz, 2H, H-3), 4.81 (ψt, *J* = 9.7 Hz, 2H, H-4'), 4.70 (d, *J* = 12.3 Hz, 2H, C$H_a$$H_b$), 4.68–4.61 (m, 4H, H-1, H-1'), 4.54 (d, *J* = 12.3 Hz, 2H, C$H_a$$H_b$), 4.35 (d, *J* = 11.0 Hz, 2H, H-6a), 4.26 (dd, *J* = 3.6, 12.1 Hz, 2H, H-6a'), 4.06 (dd, *J* = 6.0, 12.0 Hz, 2H, H-6b), 3.89 (d, *J* = 12.0 Hz, 2H, H-6b'), 3.82–3.79 (m,

2H, H-5’), 3.72 (ψt, J = 9.4 Hz, 2H, H-4), 3.65–3.54 (m, 6H, H-2, H-2’, H-5), 2.06 (s, 6H, $C(O)CH_3$), 2.00 (s, 6H, $C(O)CH_3$), 1.95 (s, 6H, $C(O)CH_3$), 1.93 (s, 6H, $C(O)CH_3$), 1.90 (s, 6H, $C(O)CH_3$), 1.75 (s, 6H, $C(O)CH_3$), 1.65 (s, 6H, $C(O)CH_3$); ^{13}C NMR (DMSO-d_6, 150.9 MHz) δ 170.0, 169.9, 169.3, 169.1, 169.0, 168.9 (each C=O), 143.4 (triazole-4), 135.8 (quaternary Ar), 128.2 (Ar), 124.1 (triazole-5), 100.2 (C-1’), 99.2 (C-1), 76.0 (C-4), 73.4 (C-3), 72.4 (C-3’), 71.9 (C-5), 70.4 (C-5’), 68.3 (C-4’), 62.5 (C-6), 61.7 (CH_2), 61.7 (C-6’), 53.6 (C-2’), 53.4 (C-2), 52.3 (N-CH_2), 22.5, 22.5, 20.8, 20.6, 20.3, 20.3, 20.2 (each CH_3); *m*/*z* (MALDI–FTICR) for $C_{66}H_{88}N_{10}O_{32}$: calcd. 1555.5458 $[M + Na]^+$; found 1555.5482.

1,4-Bis(3-{4-[2-acetamido-3,6-di-*O*-acetyl-2-deoxy-4-*O*-(2-acetamido-3,4,6-tri-*O*-acetyl-2-deoxy-β-D-glucopyranosyl)-β-D-glucopyranosyloxymethyl]-1*H*-1,2,3-triazole-1-yl}propyloxy)butane (B4)

1,4-Bis(3-aminopropyloxy)butane (**A4**, 19 mg, 0.093 mmol) and propargyl glycoside **1** (145 mg, 0.216 mmol) were reacted according to GP 1 using 15 mol % $CuSO_4$ and 2.2 equiv sodium ascorbate. After FC (chloroform/MeOH 8:1) **B4** (42 mg, 28%) was isolated as a white powder. ^1H NMR ($CDCl_3$, 600 MHz) δ 7.61 (s, 2H, triazole-5), 6.93 (d, J = 9.0 Hz, 2H, NH’), 6.75 (d, J = 9.1 Hz, 2H, NH), 5.18 (ψt, J = 9.8 Hz, 2H, H-3’), 5.07 (ψt, J = 9.2 Hz, 2H, H-3), 5.01 (ψt, J = 9.6 Hz, 2H, H-4’), 4.88 (d, J = 12.5 Hz, 2H, CH_aH_b), 4.73–4.65 (m, 4H, H-1, CH_aH_b), 4.60 (d, J = 8.3 Hz, 2H, H-1’), 4.43 (t, J = 6.7 Hz, 4H, N-$CH_2CH_2CH_2$), 4.40–4.36 (m, 4H, H-6a, H-6a’), 4.21–4.16 (m, 2H, H-6b), 4.07–3.99 (m, 4H, H-2, H-6b’), 3.87–3.80 (m, 2H, H-2’), 3.80–3.75 (m, 2H, H-4), 3.70–3.64 (m, 4H, H-5, H-5’), 3.42–3.36 (m, 8H, CH_2OCH_2), 2.14–2.08 (m, 10H, N-$CH_2CH_2CH_2$, $C(O)CH_3$), 2.05 (s, 6H, $C(O)CH_3$), 2.02 (s, 6H, $C(O)CH_3$), 1.99–1.79 (m, 12H, $C(O)CH_3$), 1.91 (s, 6H, $C(O)CH_3$), 1.83 (s, 6H, C(O)CH3), 1.61–1.58 (m, 4H, $CH_2CH_2CH_2CH_2$); ^{13}C NMR ($CDCl_3$, 150.9 MHz) δ 171.1, 171.1, 171.0, 170.8, 170.8, 170.6, 169.4 (each C=O), 123.4 (triazole-5), 101.1 (C-1’), 100.6 (C-1), 76.1 (C-4), 72.9 (C-5 or C-5’), 72.7 (C-3), 72.5 (C-3’), 71.7 (C-5 or C-5’), 70.7 ($CH_2CH_2CH_2CH_2$), 68.1 (C-4’), 66.6 (N-$CH_2CH_2CH_2$), 62.6 (CH_2), 62.5 (C-6), 61.7 (C-6’), 54.5 (C-2’), 53.3 (C-2), 47.4 (N-$CH_2CH_2CH_2$), 30.3 (N-$CH_2CH_2CH_2$), 26.3 ($CH_2CH_2CH_2CH_2$), 23.0, 22.9, 22.9, 20.9, 20.7, 20.6, 20.5 (each CH_3); *m*/*z* (MALDI–FTICR) for $C_{68}H_{100}N_{10}O_{34}$: calcd. 1623.6296 $[M + Na]^+$; found 1623.6276.

4,7,10-Trioxa-1,13-tridecanediazide (2)

4,7,10-Trioxa-1,13-tridecanediamine (**A5**, 125 µL, 0.571 mmol) was subjected to diazo transfer as described in GP 1 using 0.02 equiv $CuSO_4$. Instead of adding the compounds for the cycloaddition, the reaction mixture was diluted with water, the organic solvents were removed under reduced pressure and the remaining water was extracted three times with dichloromethane. The combined organic phases were dried over Na_2SO_4, filtered and concentrated under reduced pressure. After FC (petroleum ether/ethyl acetate 2:1) **2** (148 mg, 95%) was isolated as a colorless oil. Found: C, 44.29; H, 7.46; N, 30.72; calcd. for $C_{10}H_{20}N_6O_3$: C, 44.11; H, 7.40; N, 30.86 %; ^{1}H NMR ($CDCl_3$, 600 MHz) δ 3.65–3.62 (m, 4H, (O-$CH_2$$C$*$H_2$*$)_2$O), 3.61–3.58 (m, 4H, (O-C*H_2*$CH_2)_2$O), 3.54 (t, *J* = 6.1 Hz, 4H, N_3-CH_2CH_2C*H_2*), 3.39 (t, *J* = 6.7 Hz, 4H, N_3-C*H_2*CH_2CH_2), 1.85 (ψquin., *J* = 6.4 Hz, 4H, N_3-CH_2C*H_2*CH_2); ^{13}C NMR ($CDCl_3$, 150.9 MHz) δ 70.5 ((O-CH_2*C*$H_2)_2$O), 70.3 ((O-*C*$H_2CH_2)_2$O), 67.8 (N_3-CH_2CH_2*C*H_2), 48.4 (N_3-*C*$H_2CH_2CH_2$), 29.0 (N_3-CH_2*C*H_2CH_2); *m/z* (ESI–IT) 273.2 $[M + H]^+$, 295.2. [M + Na] $^+$, 311.2 $[M + K]^+$.

1,13-Bis{4-[2-Acetamido-3,6-di-*O*-acetyl-2-deoxy-4-*O*-(2-acetamido-3,4,6-tri-*O*-acetyl-2-deoxy-β-D-glucopyranosyl)-β-D-glucopyranosyloxymethyl]-1*H*-1,2,3-triazole-1-yl}-4,7,10-trioxatridecane (B5)

4,7,10-Trioxa-1,13-tridecanediazide (**2**, 25 mg, 0.092 mmol), propargyl glycoside **1** (236 mg, 0.350 mmol), $CuSO_4$ (1.9 mg, 0.012 mmol), TBTA (15 mg, 0.028 mmol) and sodium ascorbate (15 mg, 0.075 mmol) in 2 mL dichloromethane/methanol/water (3:10:3) were heated to 80 °C under microwave irradiation for 4 h. The reaction mixture was diluted with water and the organic solvents were removed under reduced pressure. Remaining water was removed by lyophilization. After FC (chloroform/MeOH 8:1) **B5** (76 mg, 51%) was isolated as a white powder. ^{1}H NMR (CD_3OD, 600 MHz) δ 7.99 (s, 2H, triazole-5), 5.30 (ψt, *J* = 9.9 Hz, 2H, H-3'), 5.14 (dd, *J* = 9.1, 10.2 Hz, 2H, H-3), 4.95 (ψt, *J* = 9.7 Hz, 1H, H-4'), 4.85 (d, *J* = 12.5 Hz, 2H, C*H_a*H_b), 4.81 (d, *J* = 8.4 Hz, 2H, H-1'), 4.75–4.70 (m, 4H, H-1, CH_a*H_b*), 4.59–4.56 (m, 2H, H-6a), 4.49 (t, *J* = 7.0 Hz, 4H, N-C*H_2*CH_2CH_2), 4.43 (dd, *J* = 3.9, 12.5 Hz, 2H, H-6a'), 4.09 (dd, *J* = 5.7, 12.0 Hz, 2H, H-6b), 4.03–3.99 (m, 2H, H-6b'), 3.87–3.81 (m, 6H, H-2, H-4, H-5'), 3.75–3.69 (m, 4H, H-2', H-5), 3.65–3.62 (m, 4H, O-CH_2), 3.60–3.57 (m, 4H, O-CH_2), 3.46 (t, *J* = 5.8 Hz, 4H, N-CH_2CH_2C*H_2*), 2.16–2.10 (m, 10H, N-CH_2C*H_2*CH_2, C(O)CH_3), 2.05–2.04 (m, 12H, C(O)CH_3), 1.98 (s, 6H, C(O)CH_3), 1.97

(s, 6H, C(O)CH_3), 1.94 (s, 6H, C(O)CH_3), 1.88 (s, 6H, C(O)CH_3); ^{13}C NMR (CD_3OD, 150.9 MHz) δ 173.8, 173.7, 172.4, 172.1, 172.1, 171.7, 171.2 (each C=O), 145.0 (triazole-4), 125.9 (triazole-5), 101.8 (C-1'), 101.1 (C-1), 77.5 (C-4), 74.6 (C-3), 74.1 (C-5), 73.7 (C-3'), 72.7 (C-5'), 71.5 (O-CH_2), 71.3 (O-CH_2), 69.8 (C-4'), 68.3 (N-$CH_2CH_2\mathit{C}H_2$), 63.7 (C-6), 63.2 (C-1"), 63.0 (C-6'), 56.1 (C-2'), 55.5 (C-2), 48.5 (N-$\mathit{C}H_2CH_2CH_2$), 31.3 (N-$CH_2\mathit{C}H_2CH_2$), 23.1, 22.9, 21.2, 21.0, 20.8, 20.7, 20.6 (each CH_3); *m*/*z* (MALDI–FTICR) for $C_{68}H_{100}N_{10}O_{35}$: calcd. 1639.6245 [M + Na]$^+$; found 1639.6279.

Tris(2-(4-phenyl-1*H*-1,2,3-triazole-1-yl)ethyl)amine (4) and bis(2-(4-phenyl-1*H*-1,2,3-triazole-1-yl)ethyl)amine (5)

Tris(2-aminoethyl)amine (**A6**, 37 mg, 0.25 mmol) and phenylacetylene (**3**, 90 µL, 0.83 mmol) were reacted according to GP 1 using 6 mol % $CuSO_4$ and 0.30 equiv of sodium ascorbate. Two main products were formed, which were difficult to isolate in a pure form. After FC (toluene/acetone 5:2 to 1:1), fractions each containing mainly one of these products were pooled separately and the solvent was removed under reduced pressure. Both compounds were each purified again by FC (ethyl acetate/MeOH 15:1). Title compound **4** (36 mg, 27%) was isolated as a white powder. R_f = 0.28 (ethyl acetate/MeOH 10:1); ^{1}H NMR (DMSO-d_6, 399.8 MHz) δ 8.18 (s, 3H, triazole-5), 7.71 (m, 6H, H-Ar), 7.35 (m, 6H, H-Ar), 7.29 (m, 3H, C-Ar), 4.38 (t, *J* = 6.1, 6H, N($CH_2\mathit{CH_2}$)$_3$), 3.08 (t, *J* = 6.1, 6H, N($\mathit{CH_2}CH_2$)$_3$); ^{13}C NMR (DMSO-d_6, 100.5 MHz) δ 146.1 (triazole-4), 130.6, 128.7, 127.7, 125.0 (each Ar), 121.4 (triazole-5), 53.0 (N($\mathit{C}H_2CH_2$)$_3$)), 47.7 N($CH_2\mathit{C}H_2$)$_3$); *m*/*z* (ESI–IT) 531.5 [M + H]$^+$, 553.5 [M + Na]$^+$, 569.4 [M + K]$^+$; Anal. for $C_{30}H_{30}N_{10}$: calcd. C 67.90, H 5.70, N 26.40. found C 67.45, H 5.94, N 26.22.

Title compound **5** (11 mg, 12%) was isolated as a white powder. R_f = 0.13 (ethyl acetate/MeOH 10:1); ^{1}H NMR (DMSO-d_6, 399.8 MHz) δ 8.46 (s, 2H, triazole-5), 7.80 (m, 4H, H-Ar), 7.40 (m, 4H, H-Ar), 7.31 (m, 2H, H-Ar), 4.47 (t, *J* = 6.0, 4H, N($CH_2\mathit{CH_2}$)$_3$), 3.10 (t, J = 6.0, 4H, N($\mathit{CH_2}CH_2$)$_3$); ^{13}C NMR (DMSO-d_6, 100.5 MHz) δ 146.1 (triazole-4), 130.8, 128.8, 127.6, 125.0 (each Ar), 121.5 (triazole-5), 49.3 ($CH_2\mathit{C}H_2$)$_3$), 47.9 (N($\mathit{C}H_2CH_2$)$_3$)); *m*/*z* (ESI–TOF) for $C_{20}H_{21}N_7$: calcd. 360.1931 [M + H]$^+$; found 360.1924; Anal. for $C_{20}H_{21}N_7$: calcd. C, 66.83; H, 5.89; N 27.28; found: C, 66.95; H, 5.83; N, 27.36.

Tris(2-{4-[2-acetamido-3,6-di-*O*-acetyl-2-deoxy-4-*O*-(2-acetamido-3,4,6-tri-*O*-acetyl-2-desoxy-β-D-glucopyranosyl)-β-D-glucopyranosyloxymethyl]-1*H*-1,2,3-triazole-1-yl}ethyl)amine (B6)

Tris(2-aminoethyl)amine (**A6**, 12 mg, 0.084 mmol) and propargyl glycoside **1** (203 mg, 0.302 mmol) were reacted according to GP 1 using 24 mol % $CuSO_4$ and 1.4 equiv sodium ascorbate. In this case an unknown side product was formed, which was difficult to remove from the title compound. After FC (chloroform/MeOH 5:1) followed by purification using reversed phase HPLC (25–60 % B in 20 min; t_R = 12.1 min) **B6** (12 mg, 6%) was isolated as a white powder. ^{1}H NMR ($CDCl_3$, 600 MHz) δ 7.59–6.80 (m, 9H, triazole-5, NH, NH'), 5.25–5.19 (m, 3H, H-3'), 5.16–5.07 (m, 6H, H-3, H-4'), 5.01–4.93 (m, 3H, CH_aH_b), 4.80–4.74 (m, 3H, H-1), 4.69–4.53 (m, 12H, H-1', CH_aH_b, H-6a, H-6a'), 4.30–3.70 (m, 27H, H-2, H-2', $(CH_2CH_2)_3N$, H-4, H-5, H-5', H-6b, H-6b'), 3.07–2.90 (m, 6H, $(CH_2CH_2)_3N$), 2.12 (s, 9H, $C(O)CH_3$), 2.09 (s, 9H, $C(O)CH_3$), 2.07 (s, 9H, $C(O)CH_3$), 2.04–2.00 (m, 27H, $C(O)CH_3$), 1.82 (s, 9H, $C(O)CH_3$; ^{13}C NMR ($CDCl_3$, 150.9 MHz) δ 171.8, 171.4, 170.7, 170.6, 170.4, 169.5 (each C=O), 142.9 (triazole-4), 124.9 (triazole-5), 102.3 (C-1'), 101.5 (C-1), 77.9 (C-4), 73.6 (C-3', C-5), 72.6 (C-3), 71.9 (C-5'), 67.8 (C-4'), 63.2 (CH_2), 62.6 (C-6), 61.3 (C-6'), 54.5 (C-2'), 54.4 ($(CH_2CH_2)_3N$)), 53.4 (C-2), 48.7 ($(CH_2CH_2)_3N$)), 23.2, 22.7, 20.9, 20.7, 20.6, 20.5 (each CH_3); *m*/*z* (MALDI–FTICR) for $C_{93}H_{132}N_{16}O_{48}$: calcd. 2263.8272 $[M + Na]^+$; found 2263.8521.

4-[2-Acetamido-2-deoxy-4-*O*-(2-acetamido-2-deoxy-β-D-glucopyranosyl)-β-D-glucopyranosyloxymethyl]-1-benzyl-1*H*-1,2,3-triazole (C1)

B1 (38 mg, 0.047 mmol) was deacetylated according to GP 2. **C1** (27 mg, 96%) was isolated as a white powder. ^{1}H NMR (DMSO-d_6, 600 MHz) δ 8.40 (s, 1H, triazole-5), 7.80 (d, *J* = 9.1 Hz, 1H, NH'), 7.69 (d, *J* = 8.3 Hz, 1H, NH), 7.39–7.35 (m, 2H, Ph), 7.34–7.29 (m, 3H, Ph), 5.58 (s, 2H, N-CH_2), 4.76 (d, *J* = 12.4 Hz, 1H, CH_aH_b), 4.60 (d, *J* = 12.4 Hz, 1H, CH_aH_b), 4.40 (d, *J* = 7.9 Hz, 1H, H-1), 4.34 (d, *J* = 8.5 Hz, 1H, H-1'), 3.74–3.70 (m, 1H, H-6a'), 3.66–3.62 (m, 1H, H-6a), 3.51–3.42 (m, 4H, H-2, H-2', H-6b, H-3), 3.39–3.35 (m, 1H, H-6b'), 3.30–3.24 (m, 2H, H-3', H-4), 3.20–3.14 (m, 2H, H-5, H-5'), 3.03 (ψt, J = 9.2 Hz, 1H, H-4'), 1.83 (s, 3H, $C(O)CH_3$), 1.70 (s, 3H, $C(O)CH_3$); ^{13}C NMR (DMSO-d_6, 150.9 MHz) δ 169.0 (C=O), 168.7 (C=O), 143.9 (triazole-4), 135.9, 128.7, 128.0, 127.8 (je Ph), 124.1 (triazole-5), 102.1 (C-1'), 100.0 (C-1), 81.4 (C-4), 76.8 (C-5'), 74.9 (C-5), 73.9 (C-3'), 72.4 (C-3), 70.6 (C-4'), 61.3

(CH_2), 60.9 (C-6'), 60.1 (C-6), 55.3 (C-2'), 54.3 (C-2), 52.7 (N-CH_2), 22.9 (CH_3), 22.9 (CH_3); *m*/*z* (MALDI–FTICR) for $C_{26}H_{37}N_5O_{11}$: calcd. 618.2382 $[M + Na]^+$; found 618.2380.

α,α' –Bis{4-[2-acetamido-2-deoxy-4-*O*-(2-acetamido-2-deoxy-β-D-glucopyranosyl)-β-D-glucopyranosyloxymethyl]-1*H*-1,2,3-triazole-1-yl}-*m*-xylylene (C2)

B2 (41 mg, 0.027 mmol) was deacetylated according to GP 2. **C2** (29 mg, 97%) was isolated as a white powder. ^1H NMR (DMSO-d_6, 600 MHz) δ 8.05 (s, 2H, triazole-5), 7.82 (d, J = 9.1 Hz, 2H, NH'), 7.71 (d, J = 8.3 Hz, 2H, NH), 7.36 (t, J = 7.7 Hz, 1H, H-Ar5), 7.31 (br. s, 1H, H-Ar2), 7.25–7.22 (m, 2H, H-Ar4, H-Ar6), 5.57 (s, 4H, N-CH_2), 4.76 (d, J = 12.4 Hz, 2H, CH_aH_b), 4.60 (d, J = 12.4 Hz, 2H, CH_aH_b), 4.40 (d, J = 7.7 Hz, 2H, H-1), 4.34 (d, J = 8.5 Hz, 2H, H-1'), 3.72 (br. d, J = 10.7 Hz, 2H, H-6'a), 3.64 (dd, J = 11.2 Hz, 2H, H-6a), 3.52–3.42 (m, 8H, H-2, H-2', H-6b, H-3), 3.29–3.23 (m, 6H, H-3', H-4, H-6b'), 3.20–3.14 (m, 4H, H-5, H-5'), 3.03 (ψt, J = 9.2 Hz, 2H, H-4'), 1.83 (s, 6H, C(O)CH_3), 1.71 (s, 6H, C(O)CH_3); ^{13}C NMR (DMSO-d_6, 150.9 MHz) δ 169.0 (C=O), 168.7 (C=O), 143.8 (triazole-6), 136.5 (Ar-1, Ar-3), 129.2 (Ar-5), 127.6 (Ar-4, Ar-6), 127.4 (Ar-2), 124.2 (triazole-5), 102.1 (C-1'), 100.0 (C-1), 81.4 (C-4), 76.8 (C-5'), 74.9 (C-5), 73.9 (C-3'), 72.4 (C-3), 70.6 (C-4'), 61.2 (CH_2), 60.9 (C-6'), 59.9 (C-6), 55.3 (C-2'), 54.3 (C-2), 52.4 (N-CH_2), 22.9 (CH_3), 22.9 (CH_3); *m*/*z* (MALDI–FTICR) for $C_{46}H_{68}N_{10}O_{22}$: calcd. 1135.4402 $[M + Na]^+$; found 1135.4426.

α,α' –Bis{4-[2-acetamido-2-deoxy-4-*O*-(2-acetamido-2-deoxy-β-D-glucopyranosyl)-β-D-glucopyranosyloxymethyl]-1*H*-1,2,3-triazole-1-yl}-*p*-xylylene (C3)

B3 (50 mg, 0.033 mmol) was deacetylated according to GP 2. **C3** (37 mg, quant.) was isolated as a white powder. ^1H NMR (D_2O, 600 MHz) δ 7.90 (s, 2H, triazole-5), 7.26 (s, 4H, H-Ar), 5.51 (d, J = 15.3 Hz, 2H, N-CH_aH_b), 5.48 (d, J = 15.2 Hz, 2H, N-CH_aH_b), 4.76 (d, J = 13.1 Hz, 2H, CH_aH_b), 4.66–4.62 (m, 2H, CH_aH_b), 4.45 (d, J = 8.4 Hz, 2H, H-1'), 4.43–4.41 (m, 2H, H-1), 3.78 (br. d, J = 12.2 Hz, 2H, H-6a'), 3.69 (br. d, J = 11.6 Hz, 2H, H-6a), 3.64–3.59 (m, 4H, H-2', H-6b'), 3.58–3.50 (m, 6H, H-2, H-3, H-6b), 3.49–3.42 (m, 4H, H-3', H-4), 3.39–3.31 (m, 6H, H-4', H-5, H-5'), 1.95 (s, 6H, C(O)CH_3), 1.59 (s, 6H, C(O)CH_3); ^{13}C NMR (D_2O, 150.9 MHz) δ 174.5 (C=O), 174.2 (C=O), 143.9 (triazole-4), 135.3 (quaternary Ar), 128.9 (Ar), 125.2 (triazole-5),

101.4 (C-1’), 100.2 (C-1), 79.3 (C-4), 75.9 (C-5’), 74.5 (C-5), 73.4 (C-3’), 72.2 (C-3), 69.7 (C-4’), 62.0 (CH_2), 60.5 (C-6’), 60.1 (C-6), 55.6 (C-2’), 54.8 (C-2), 53.5 (N-CH_2), 22.1 (CH_3), 21.9 (CH_3); *m*/*z* (MALDI–FTICR) for $C_{46}H_{68}N_{10}O_{22}$: calcd. 1135.4402 $[M + Na]^+$; found 1135.4394.

1,4-Bis(3-{4-[2-acetamido-2-deoxy-4-*O*-(2-acetamido-2-deoxy-β-D-glucopyranosyl)-β-D-glucopyranosyloxymethyl]-1*H*-1,2,3-triazole-1-yl}propyloxy)butane (C4)

B4 (42 mg, 0.026 mmol) was deacetylated according to GP 2. **C4** (31 mg, 90%) was isolated as a white powder. ^{1}H NMR (D_2O, 600 MHz) δ 7.88 (s, 2H, triazole-5), 4.78 (d, J = 12.9 Hz, 2H, CH_aH_b), 4.68–4.64 (m, 2H, CH_aH_b), 4.47–4.44 (m, 4H, H-1, H-1’), 4.40 (t, J = 6.8 Hz, 4H, N-$CH_2CH_2CH_2$), 3.78 (dd, J = 2.0, 12.3 Hz, 2H, H-6a’), 3.73 (dd, J = 2.0, 12.0 Hz, 2H, H-6a), 3.64–3.59 (m, 4H, H-2’, H-6b’), 3.57–3.52 (m, 6H, H-2, H-3, H-6b), 3.50–3.46 (m, 2H, H-4), 3.43 (dd, J = 8.5, 10.4 Hz, 2H, H-3’), 3.40–3.31 (m, 14H, H-4’, N-$CH_2CH_2CH_2$, H-5, H-5’, $CH_2CH_2CH_2CH_2$), 2.05 (ψquin., J = 6.4 Hz, 4H, N-$CH_2CH_2CH_2$), 1.95 (s, 6H, C(O)CH_3), 1.79 (s, 6H, C(O)CH_3), 1.43–1.39 (m, 4H, $CH_2CH_2CH_2CH_2$); ^{13}C NMR (D_2O, 150.9 MHz) δ 175.5 (C=O), 175.3 (C=O), 126.3 (triazole-5), 102.5 (C-1’), 101.1 (C-1), 80.4 (C-4), 76.9 (C-5’), 75.6 (C-5), 74.5 (C-3’), 73.3 (C-3), 71.4 (N-$CH_2CH_2CH_2$), 70.7 (C-4’), 67.9 ($CH_2CH_2CH_2CH_2$), 63.0 (CH_2), 61.5 (C-6’), 61.1 (C-6), 56.6 (C-2’), 55.9 (C-2), 48.6 (N-$CH_2CH_2CH_2$), 30,2 (N-$CH_2CH_2CH_2$) 26.3 ($CH_2CH_2CH_2CH_2$), 23.1 (CH_3), 23.1 (CH_3); *m*/*z* (MALDI–FTICR) for $C_{48}H_{80}N_{10}O_{24}$: calcd. 1203.5239 $[M + Na]^+$; found 1203.5214.

1,13-Bis{4-[2-acetamido-2-deoxy-4-*O*-(2-acetamido-2-deoxy-β-D-glucopyranosyl)-β-D-glucopyranosyloxymethyl]-1*H*-1,2,3-triazole-1-yl}-4,7,10-trioxatridecane (C5)

B5 (74 mg, 0.046 mmol) was deacetylated according to GP 2. **C5** (58 mg, quant.) was isolated as a white powder. ^{1}H NMR (D_2O/CD_3OD 9:1, 600 MHz) δ 7.86 (s, 2H, triazole-5), 4.76 (d, J = 12.8 Hz, 2H, CH_aH_b), 4.63 (d, J = 12.9 Hz, 2H, CH_aH_b), 4.45–4.41 (m, 4H, H-1, H-1’), 4.37 (t, J = 6.8 Hz, 4H, N-$CH_2CH_2CH_2$), 3.78–3.74 (dd, J = 12.2 Hz, 2H, H-6a’), 3.72–3.69 (dd, J = 12.0 Hz, 2H, H-6a), 3.61–3.56 (m, 4H, H-2’, H-6b’), 3.56–3.28 (m, 28H, H-2, H-3, H-3’, H-4, H-4’, H-5, H-5’, H-6b, all O-CH_2), 2.02 (ψquin., J = 6.4 Hz, 4H, N-$CH_2CH_2CH_2$), 1.93 (s, 6H, C(O)CH_3), 1.77 (s, 6H, C(O)CH_3; ^{13}C NMR (D_2O/CD_3OD 9:1, 150.9 MHz) δ 175.4 (C=O), 175.2 (C=O),

144.6 (triazole-4), 126.3 (triazole-5), 102.5 (C-1’), 101.1 (C-1), 80.5 (C-4), 77.0 (C-5’), 75.6 (C-5), 74.5 (C-3’), 73.4 (C-3), 70.8 (C-4’), 70.6 (O-CH_2), 70.4 (O-CH_2), 68.2 (N-CH_2CH_2*C*H_2), 62.9 (CH_2), 61.6 (C-6’), 61.2 (C-6), 56.6 (C-2’), 55.9 (C-2), 48.4 (N-*C*$H_2CH_2CH_2$), 30.3 (N-CH_2*C*H_2CH_2), 23.1 (CH_3), 23.1 (CH_3); *m*/*z* (MALDI–FTICR) for $C_{48}H_{80}N_{10}O_{25}$: calcd. 1219.5188 $[M + Na]^+$; found 1219.5201.

Tris(2-{4-[2-acetamido-2-deoxy-4-*O*-(2-acetamido-2-desoxy-β-D-glucopyranosyl)-β-D-glucopyranosyloxymethyl]-1*H*-1,2,3-triazole-1-yl}ethyl)amine (C6)

B6 (12 mg, 0.0053 mmol) was deacetylated according to GP 2. **C6** (8 mg, 93%) was isolated as a white powder. ^1H NMR (DMSO-d_6, 600 MHz) δ 7.86 (s, 3H, triazole-5), 7.82 (d, *J* = 9.1 Hz, 3H, NH’), 7.72 (d, *J* = 8.7 Hz, 3H, NH), 4.76 (d, *J* = 12.3 Hz, 3H, CH_aH_b), 4.57 (d, *J* = 12.3 Hz, 3H, CH_aH_b), 4.40 (d, *J* = 8.1 Hz, 3H, H-1), 4.34 (d, *J* = 8.5 Hz, 3H, H-1’), 4.30–4.26 (br. t, 6H, $(CH_2CH_2)_3N$), 3.72 (br. d, *J* = 10.4 Hz, 3H, H-6a’), 3.66 (br. d, *J* = 11.2 Hz, 3H, H-6a), 3.54–3.43 (m, 12H, H-2, H-2’, H-3, H-6b), 3.39–3.25 (m, 9H, H-3’, H-4, H-6b’), 3.22–3.15 (m, 6H, H-5, H-5’), 3.03 (ψt, *J* = 9.2 Hz, 3H, H-4’), 2.96 (br. t, 6H, $(CH_2CH_2)_3N$), 1.83 (s, 9H, C(O)CH_3), 1.74 (s, 9H, C(O)CH_3); ^{13}C NMR (DMSO-d_6, 150.9 MHz) δ 169.1 (C=O), 168.8 (C=O), 143.3 (triazole-4), 124.4 (triazole-5), 102.1 (C-1’), 99.9 (C-1), 81.4 (C-4), 76.8 (C-5’), 74.9 (C-5), 73.9 (C-3’), 72.5 (C-3), 70.6 (C-4’), 61.2 (CH_2), 60.9 (C-6’), 60.0 (C-6), 55.3 (C-2’), 54.3 (C-2), 52.9 ($(CH_2CH_2)_3N$), 47.3 ($(CH_2CH_2)_3N$), 22.9 (CH_3), 22.9 (CH_3); *m*/*z* (MALDI–FTICR) for $C_{63}H_{102}N_{16}O_{33}$: calcd. 1633.6687 $[M + Na]^+$; found 1633.6654.

Determination of binding potencies of WGA ligands

Binding potencies of WGA ligands were determined according to the protocol for an enzyme-linked lectin assay (ELLA) employing covalently modified microtiter plates as described [12].

Molecular modeling

Modeling of binding modes was carried out within SYBYL 7.2 (Tripos Inc.) employing the Tripos force field [13]. Starting point for all modeling studies was the crystal structure of WGA3 in complex with a divalent ligand (PDB ID: 2X52) [14]. The binding mode of *N*,*N*'-diacetylchitobiose was taken from the crystal structure of WGA3 with one primary binding site (B2C1) occupied by *N*,*N*'-diacetylchitobiose (PDB ID: 1K7U) [15]. The conformation of this chitobiose moiety was transferred to the adjacent primary binding site (C1B2). Hydrogen atoms were added to sugars and protein, and the generated complex was energy minimized in vacuo. This minimization led to minimal changes of the bound conformation of both chitobiose moieties. Both monosaccharide units form contacts to aromatic residues and are further stabilized by polar residues of the other polypeptide chain of the WGA homodimer.

The linker structures were attached to the chitobiose moieties by using SYBYL's sketching tool, taking special care over the correct assignment of atom types. Initial structures were then minimized until convergence was reached (usually 1000 to 4000 iterations). If necessary, the linker was subjected to a short molecular dynamics simulation (up to 10 ps at 300 K) and subsequently minimized. All force field calculations were carried out without charges. Figures were prepared with Pymol (DeLano Scientific LLC).

References

1. Merkel, L.; Beckmann, H. S. G.; Wittmann, V.; Budisa, N. *ChemBioChem* **2008**, *9*, 1220-1224.
2. Hassner, A.; Stern, M. *Angew. Chem., Int. Ed. Engl.* **1986**, *25*, 478-479.
3. Hassner, A.; Stern, M.; Gottlieb, H. E. *J. Org. Chem.* **1990**, *55*, 2304-2306.
4. Dharanipragada, R.; VanHulle, K.; Bannister, A.; Bear, S.; Kennedy, L.; Hruby, V. J. *Tetrahedron* **1992**, *48*, 4733-4748.
5. Bretherick, L. *Chem. Eng. News* **1986**, *64 (Dec. 22)*, 2.
6. Peet, N. P.; Weintraub, P. M. *Chem. Eng. News* **1993**, *71 (Apr. 19)*, 4.
7. Hruby, V. J.; Boteju, L.; Li, G. *Chem. Eng. News* **1993**, *71 (Oct. 11)*, 2.
8. Beckmann, H. S. G.; Wittmann, V. *Org. Lett.* **2007**, *9*, 1-4.
9. Nyffeler, P. T.; Liang, C.-H.; Koeller, K. M.; Wong, C.-H. *J. Am. Chem. Soc.* **2002**, *124*, 10773-10778.
10. Lundquist, J. T.; Pelletier, J. C. *Org. Lett.* **2001**, *3*, 781-783.
11. Chan, T. R.; Hilgraf, R.; Sharpless, K. B.; Fokin, V. V. *Org. Lett.* **2004**, *6*, 2853-2855.

12. Maierhofer, C.; Rohmer, K.; Wittmann, V. *Bioorg. Med. Chem.* **2007**, *15*, 7661-7676.
13. Clark, M.; Cramer, R. D., III; Van Opdenbosch, N. *J. Comput. Chem.* **1989**, *10*, 982-1012.
14. Schwefel, D.; Maierhofer, C.; Beck, J. G.; Seeberger, S.; Diederichs, K.; Möller, H. M.; Welte, W.; Wittmann, V. *J. Am. Chem. Soc.* **2010**, *132*, 8704-8719.
15. Muraki, M.; Ishimura, M.; Harata, K. *Biochim. Biophys. Acta* **2002**, *1569*, 10-20.

HPLC chromatograms

B1: 20–75% B in 20 min

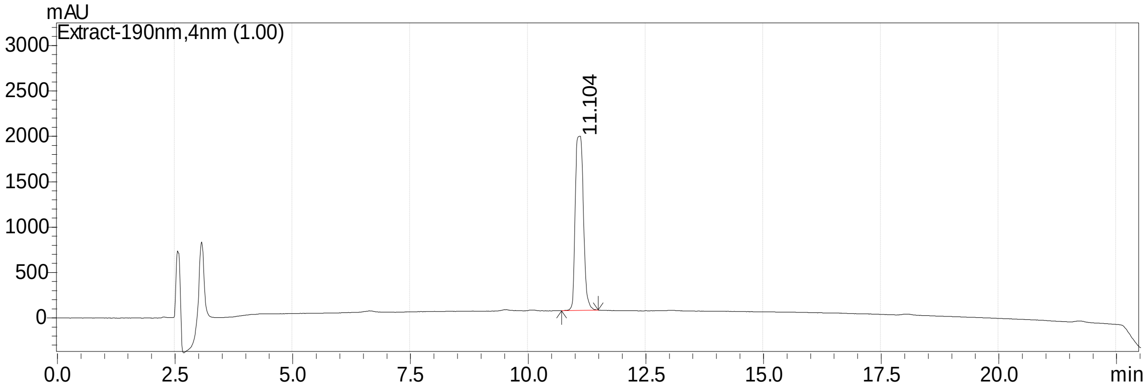

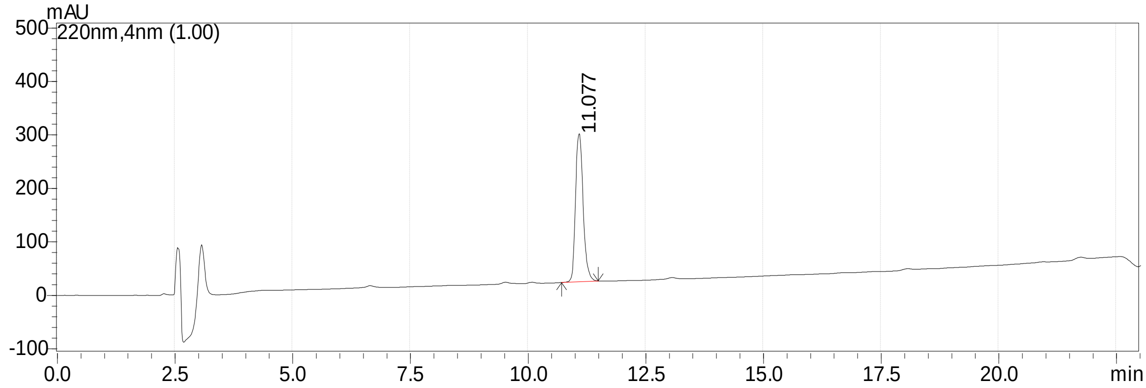

B2: 20–75% B in 20 min

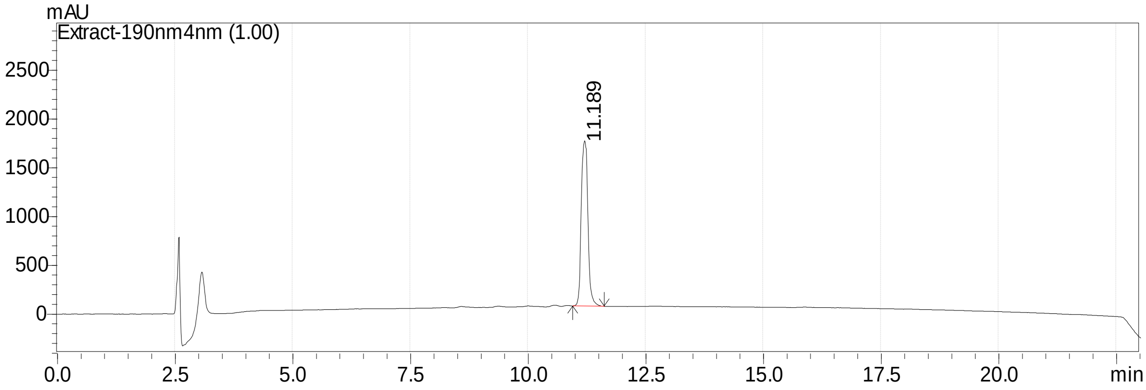

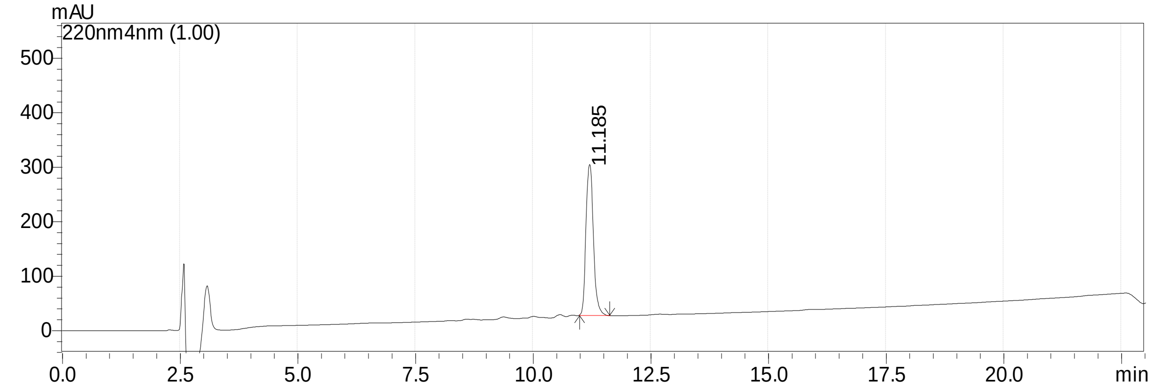

B3: 20–75% B in 20 min

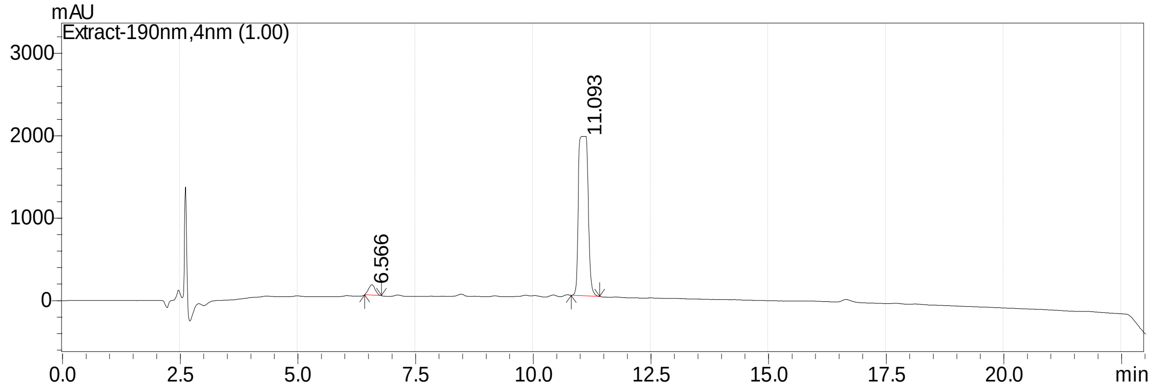

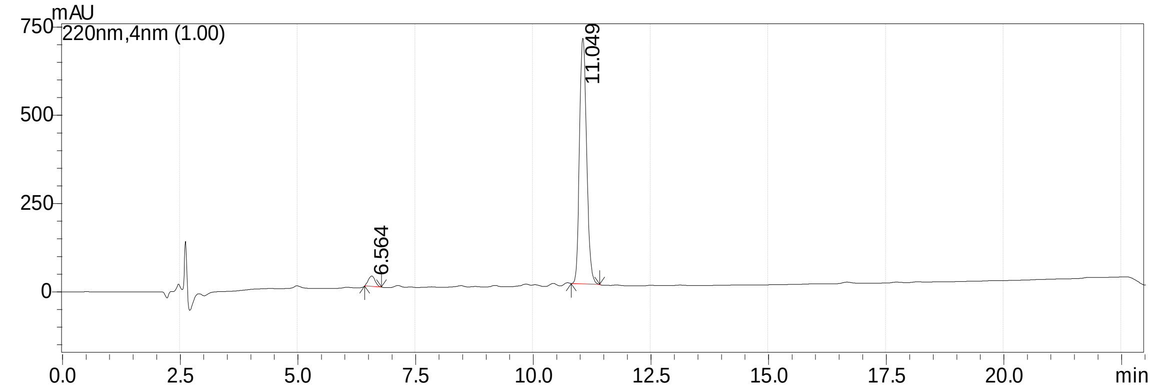

B4: 20–75% B in 20 min

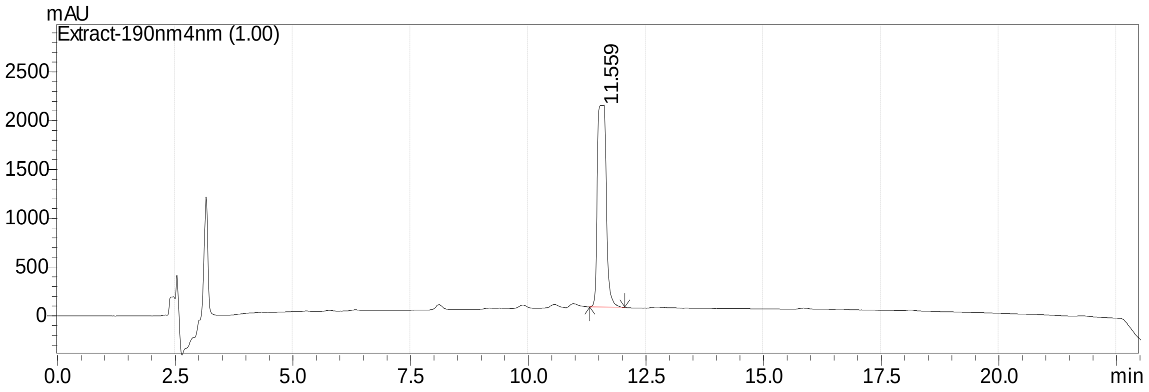

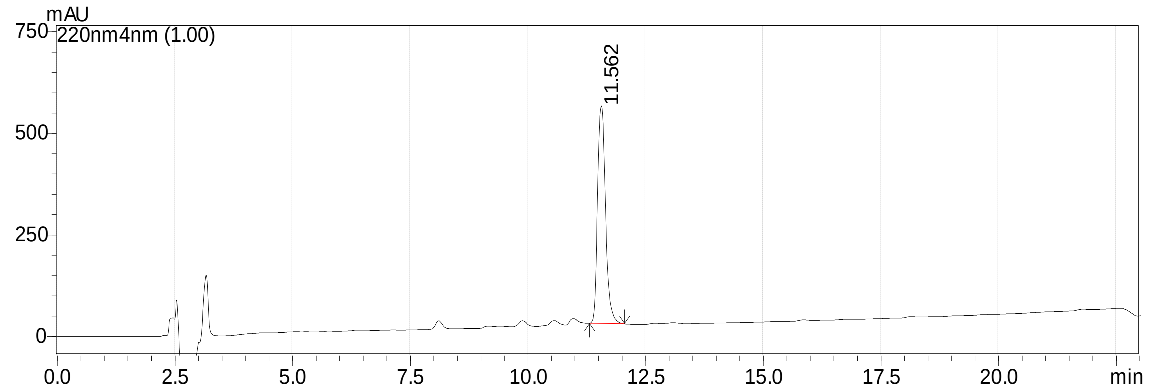

B5: 20–75% B in 20 min

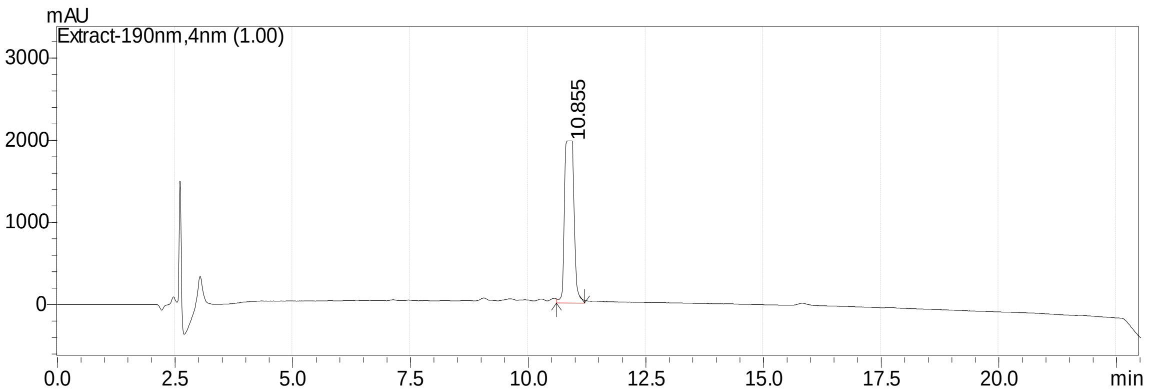

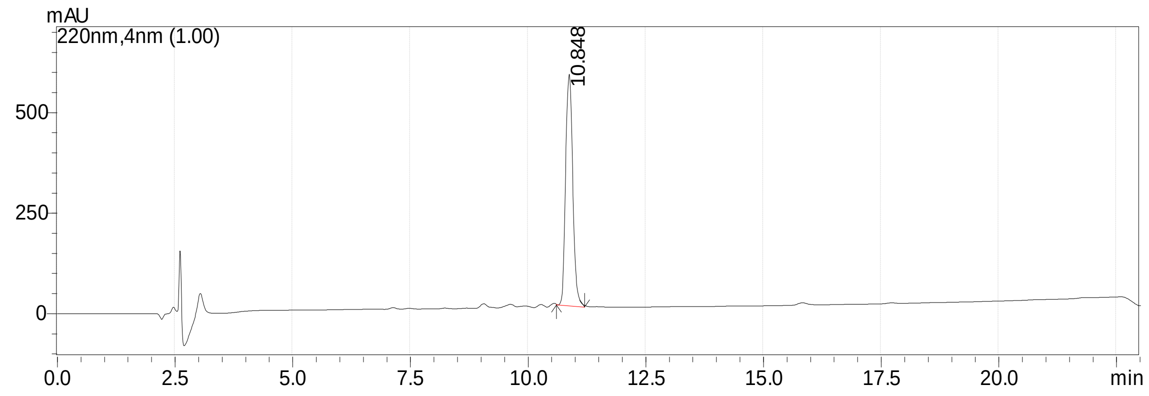

B6: 25–60% B in 20 min

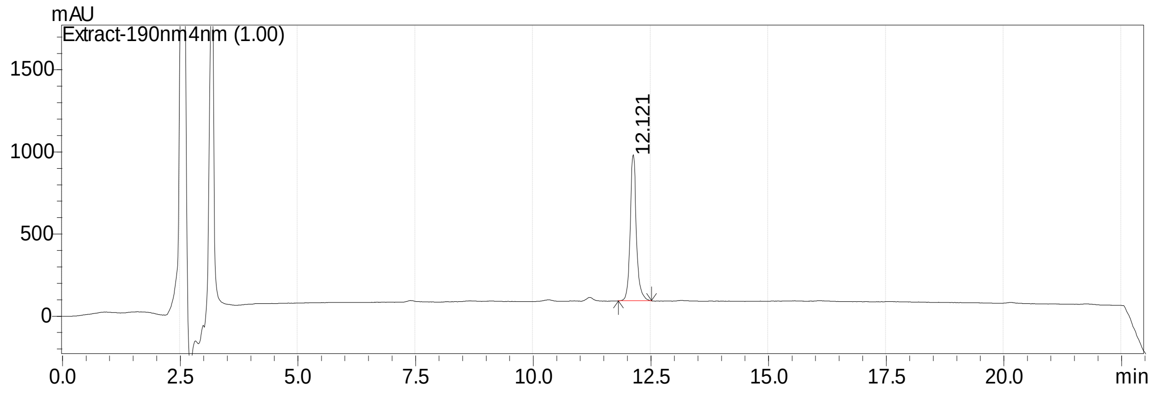

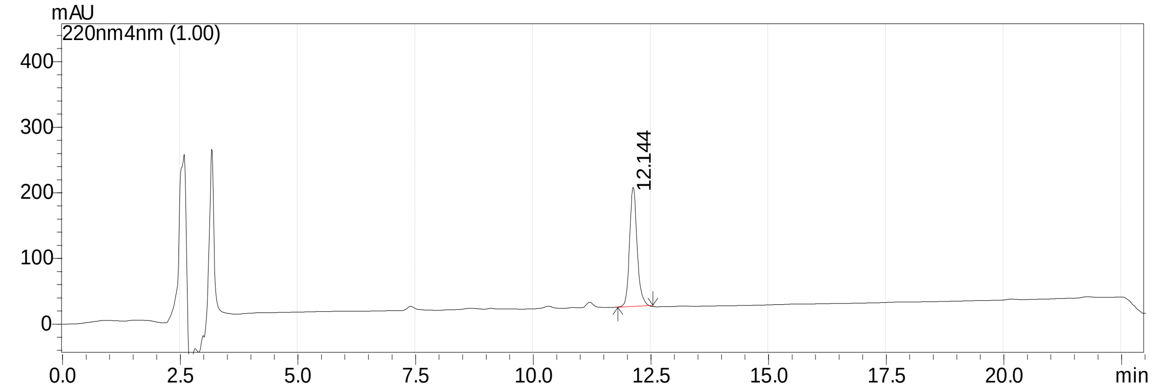

C1: 5–60% B in 20 min

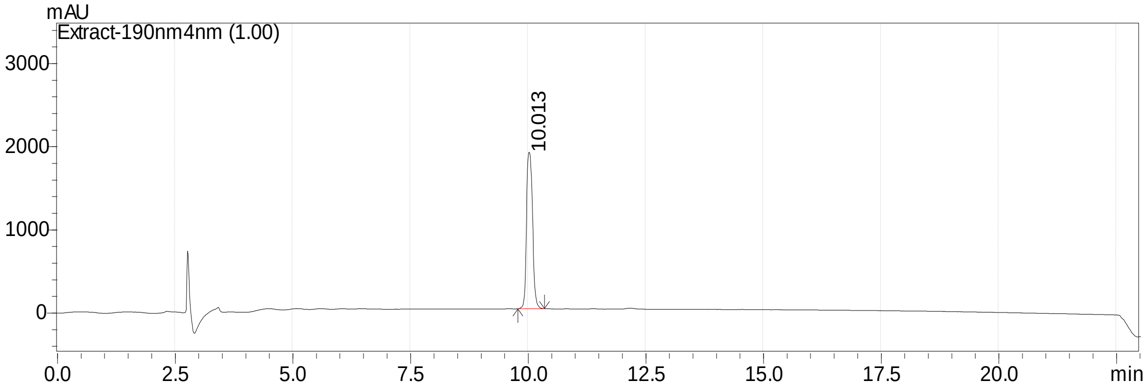

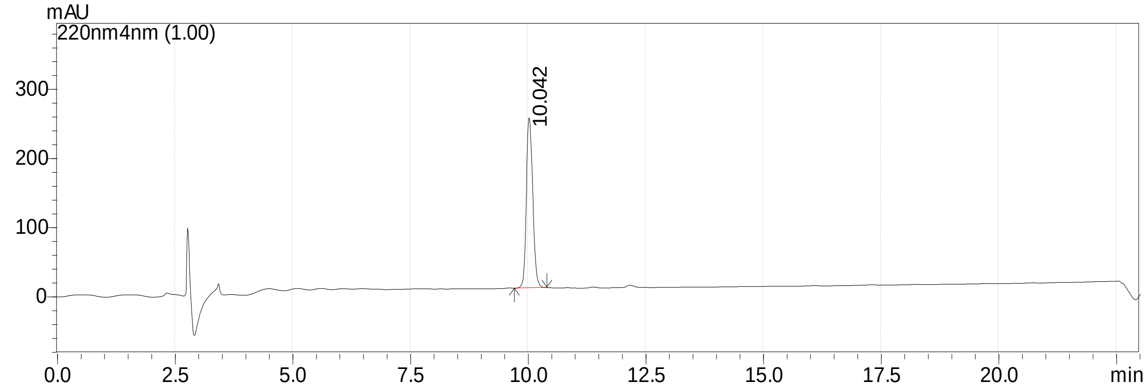

C2: 5–60% B in 20 min

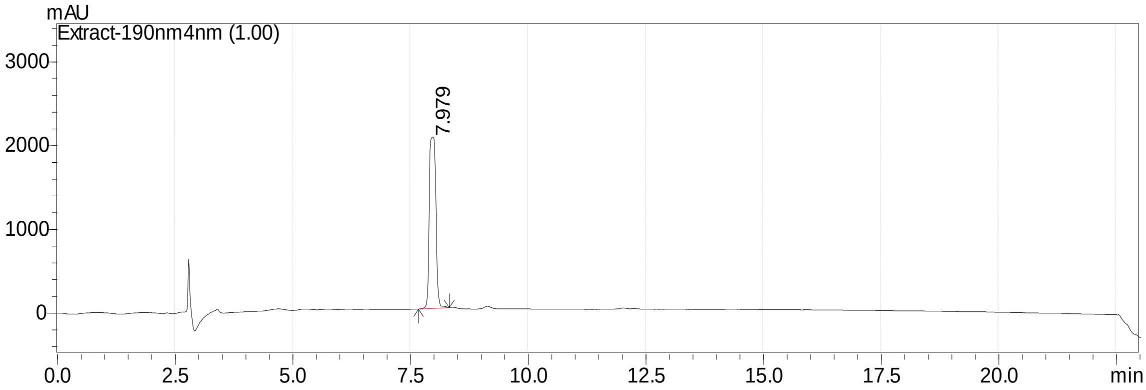

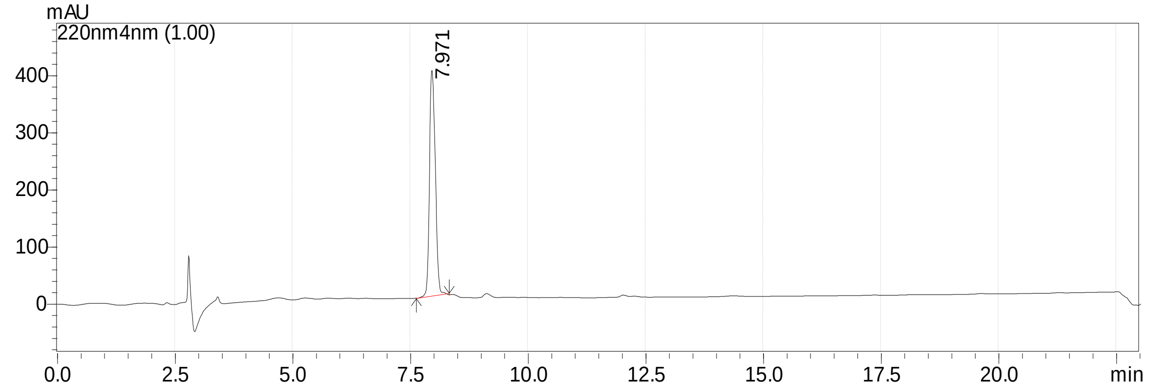

C3: 5–60% B in 20 min

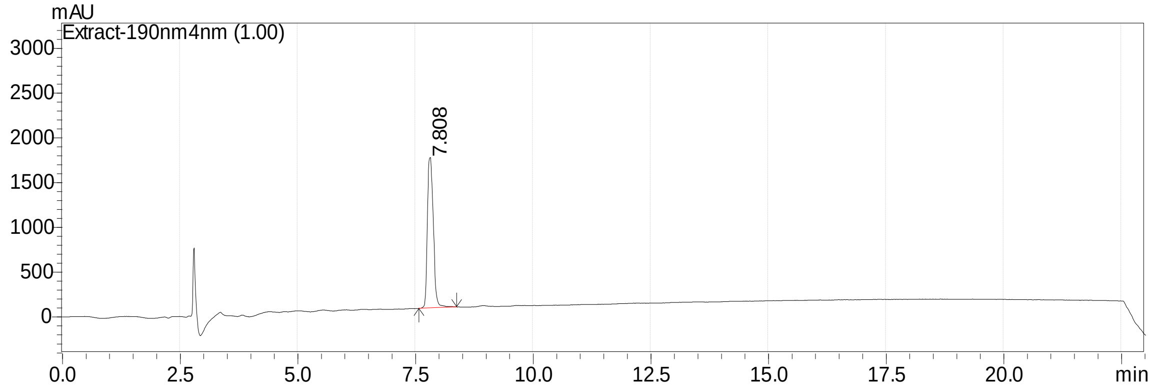

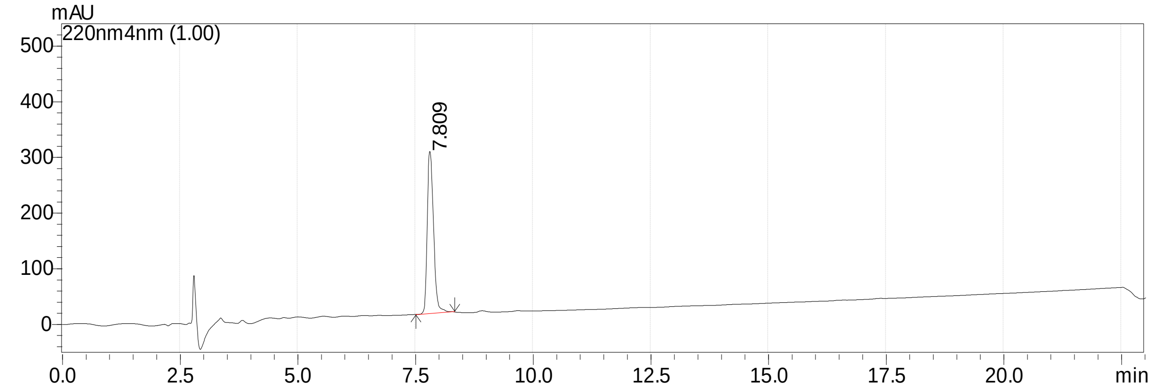

C4: 5–60% B in 20 min

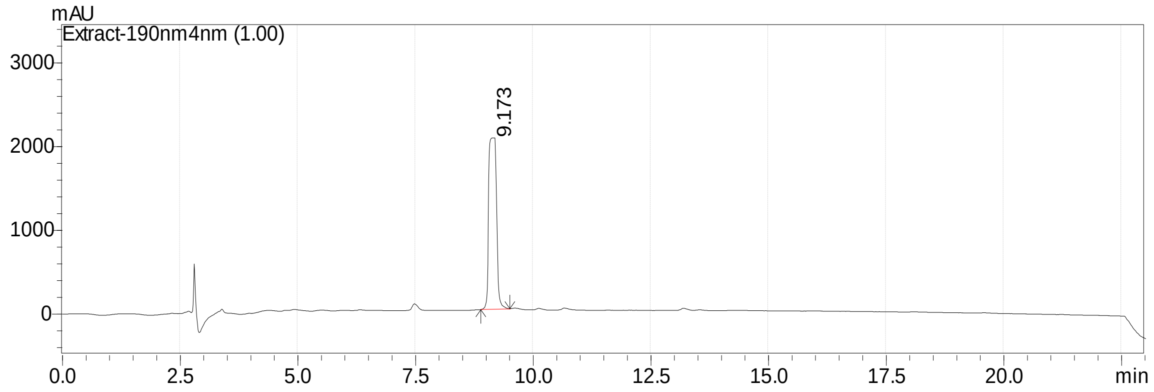

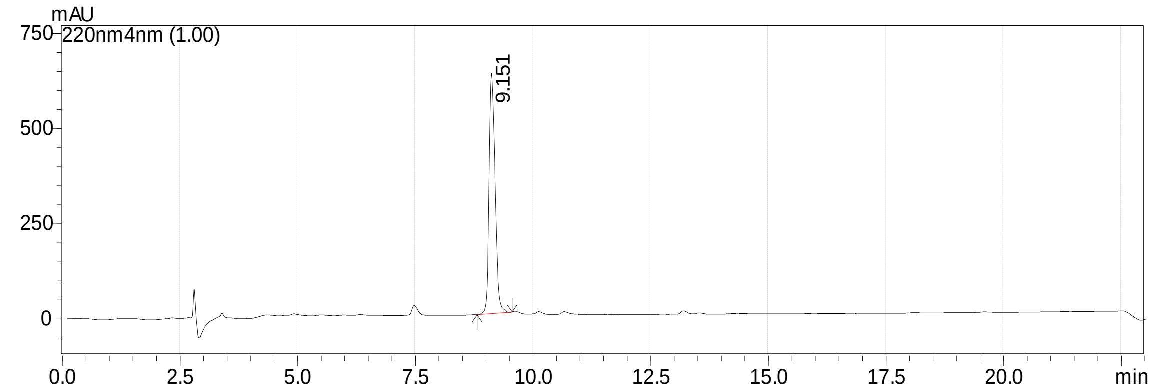

C5: 5–60% B in 20 min

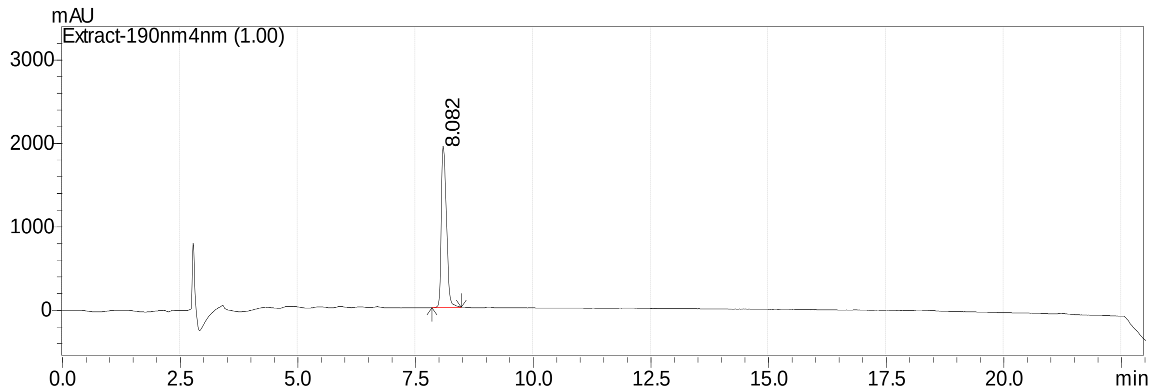

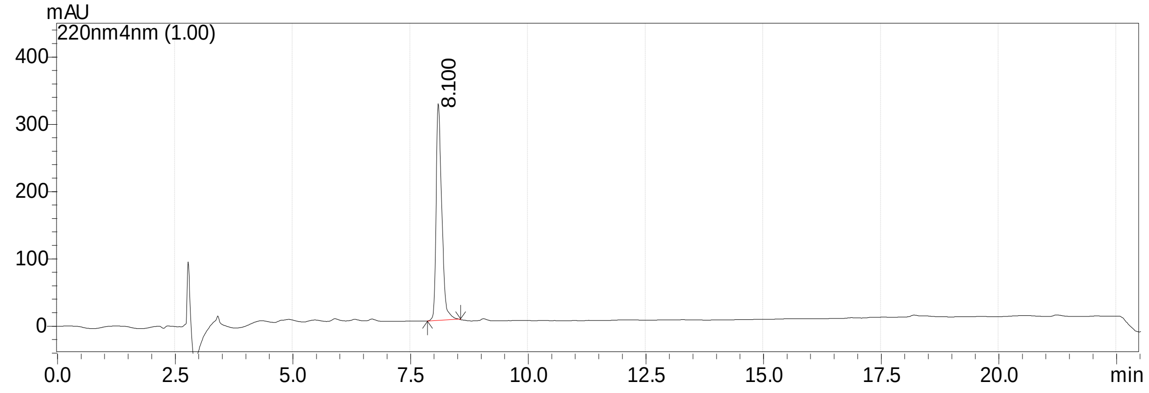

C6: 5–60% B in 20 min

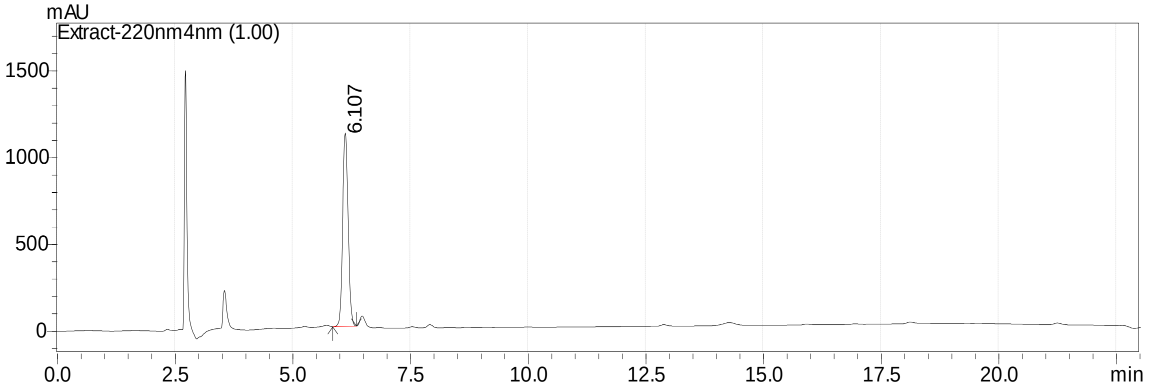

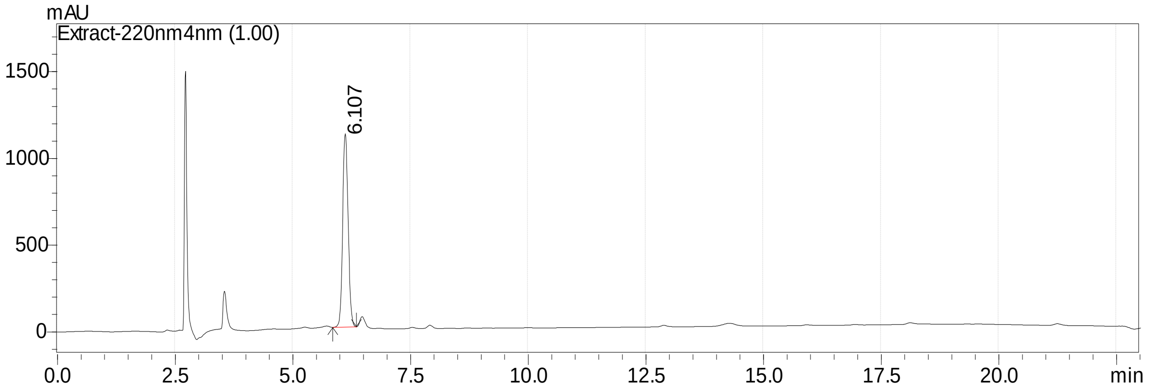

^{1}H NMR spectra

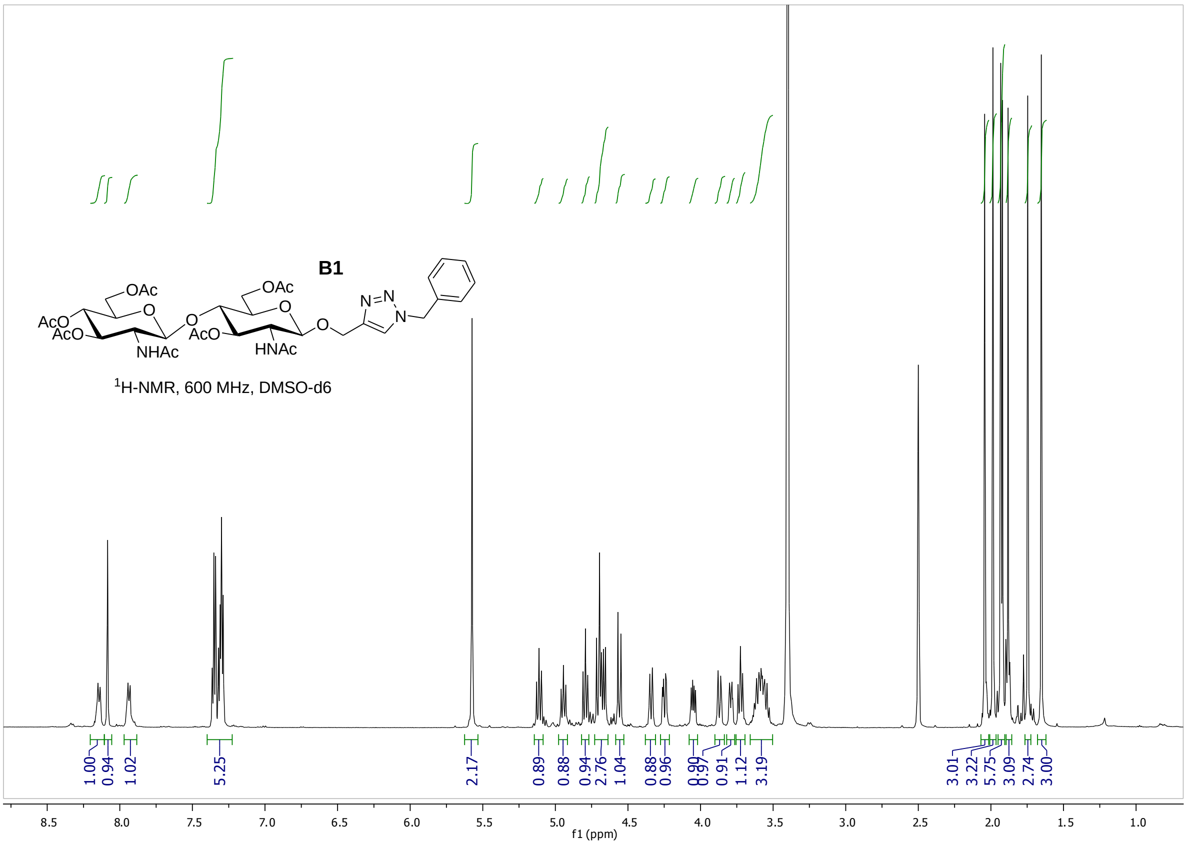

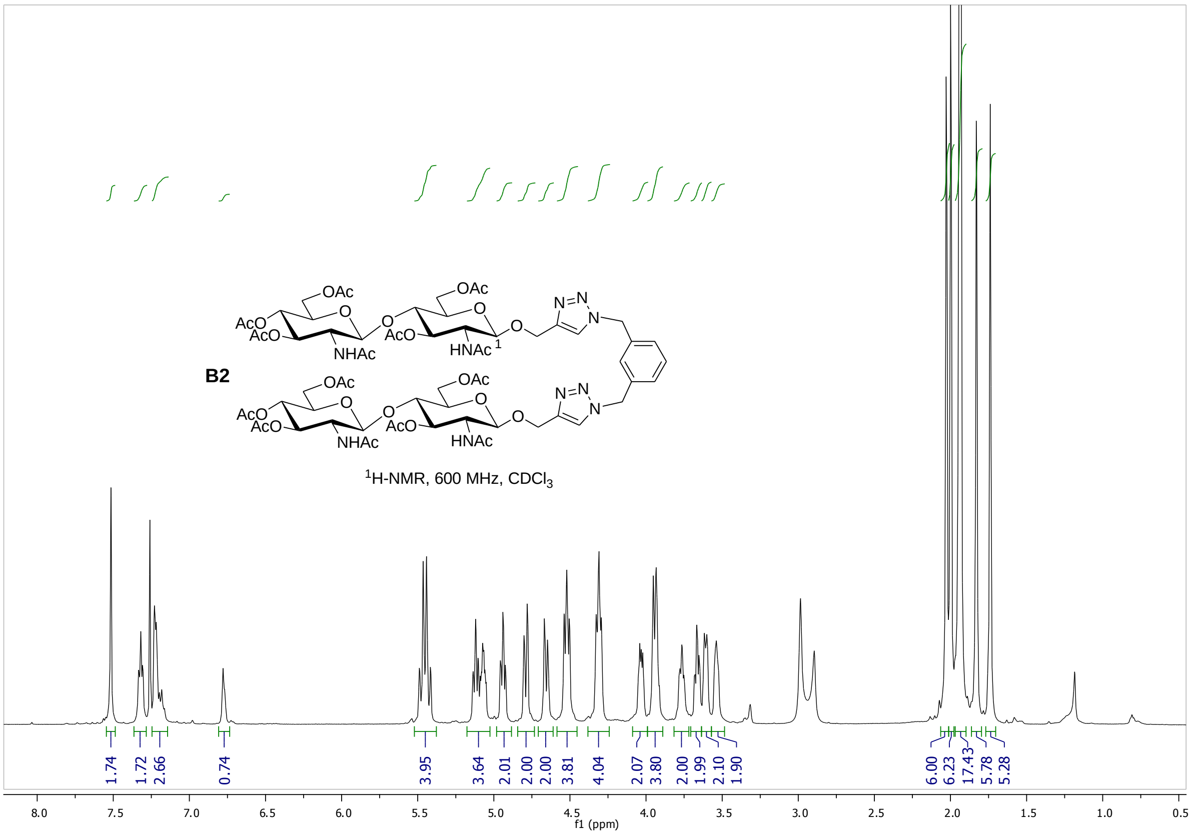

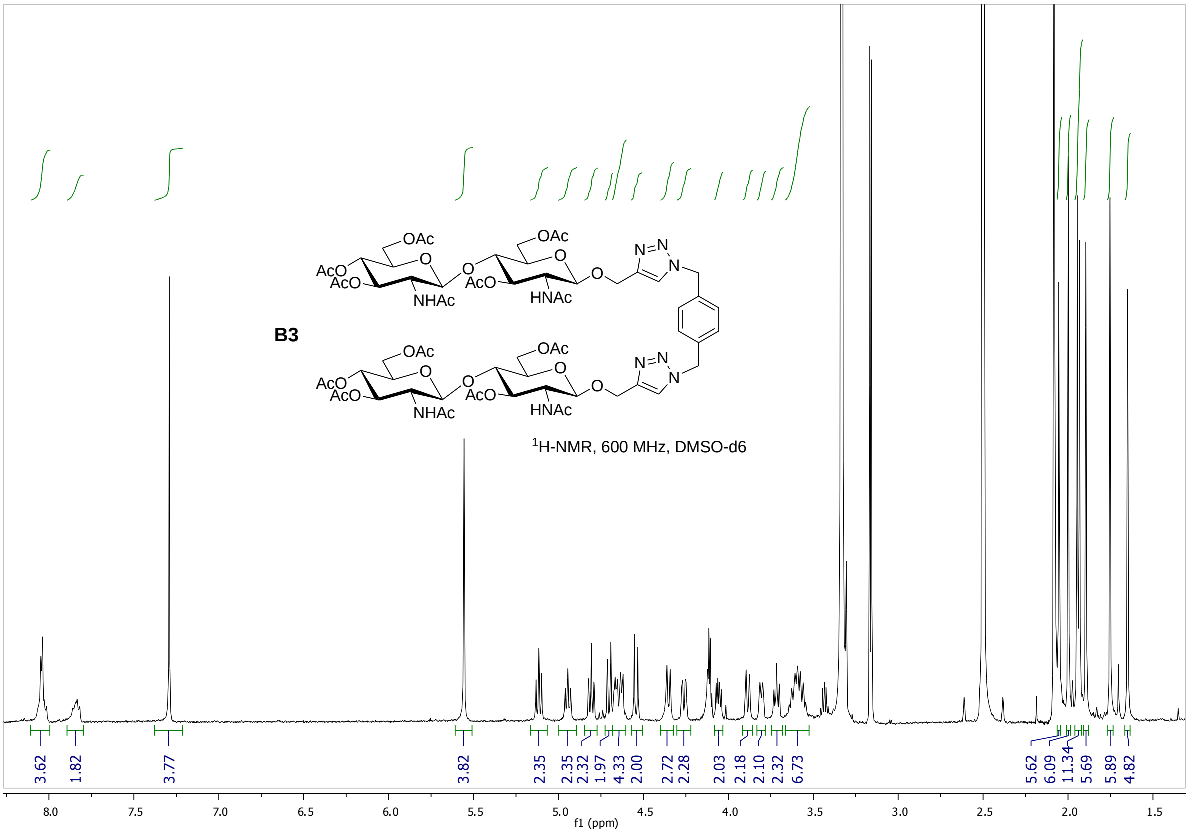
B3
OAc
AcO
NHAc
HNAc
N=N
N
O
^{1}H-NMR, 600 MHz, DMSO-d6
f1 (ppm)

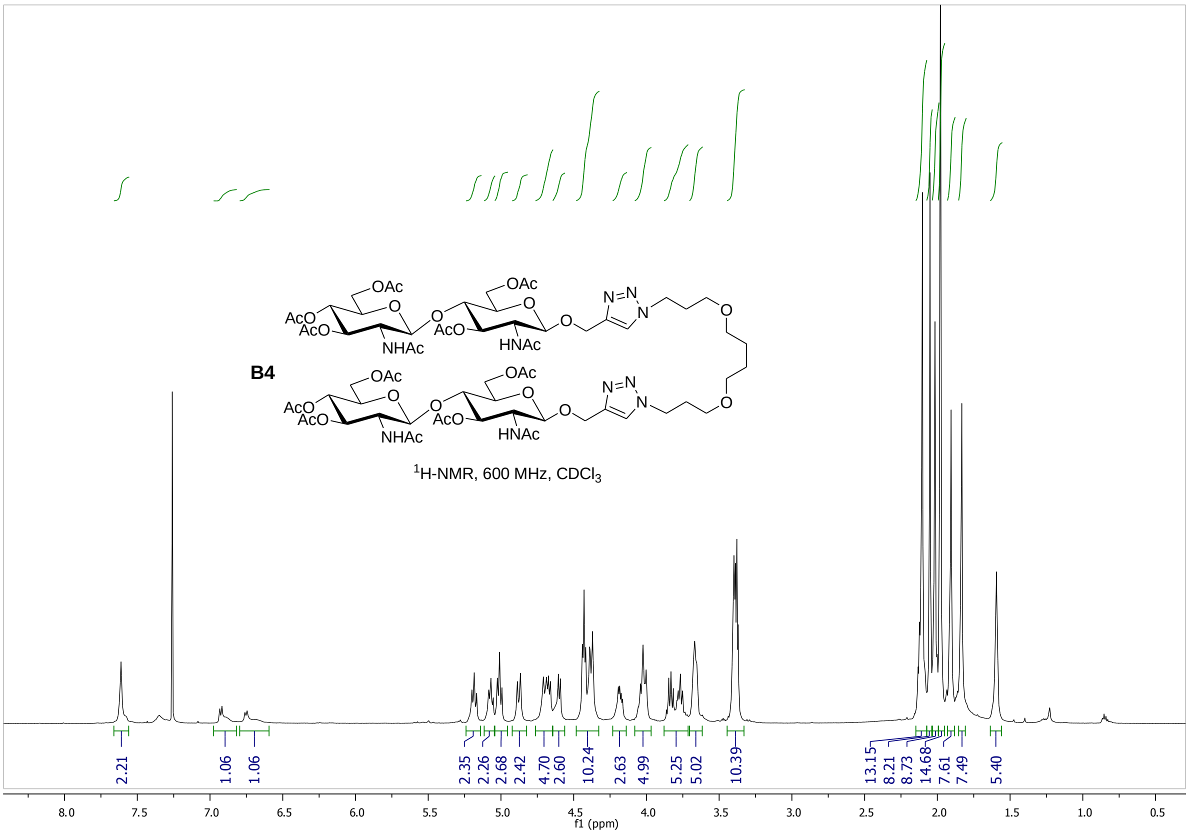
B4
OAc
AcO
NHAc
HNAc
N=N
N
O
^{1}H-NMR, 600 MHz, $CDCl_3$
f1 (ppm)

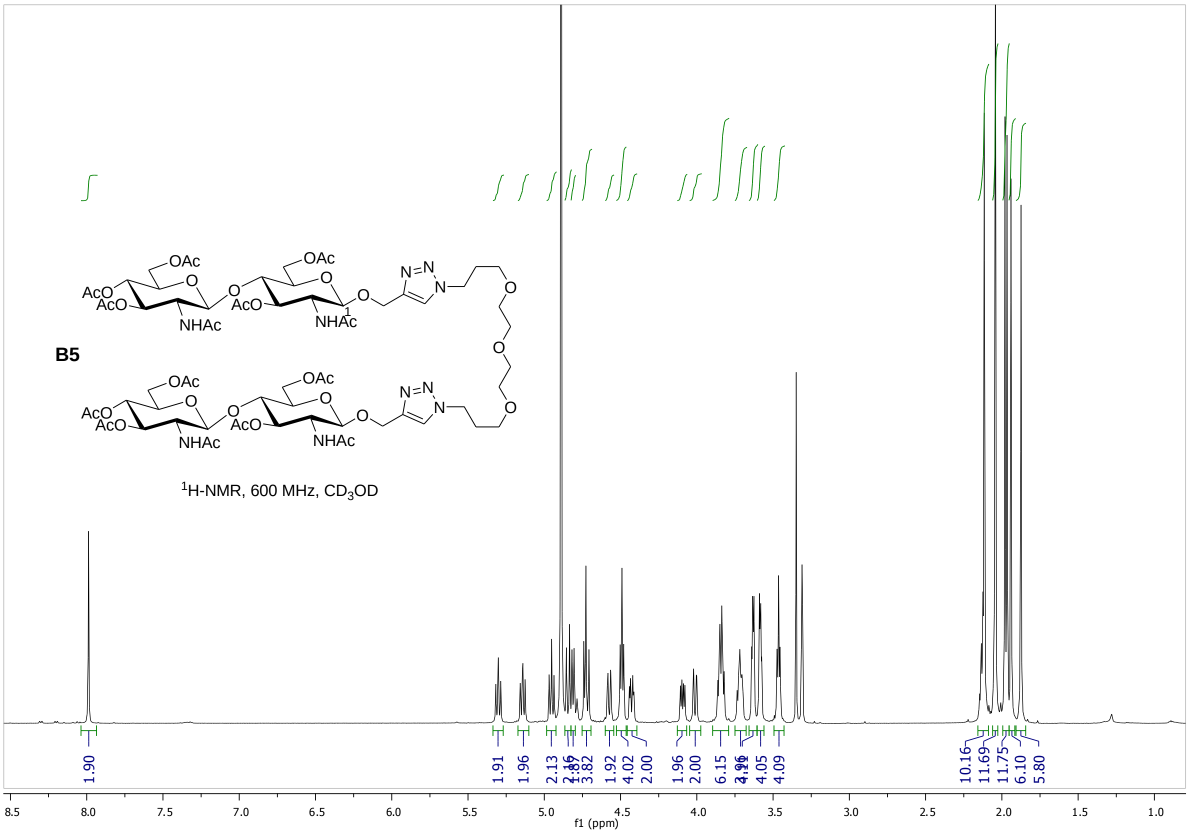
B5
¹H-NMR, 600 MHz, CD₃OD
f1 (ppm)

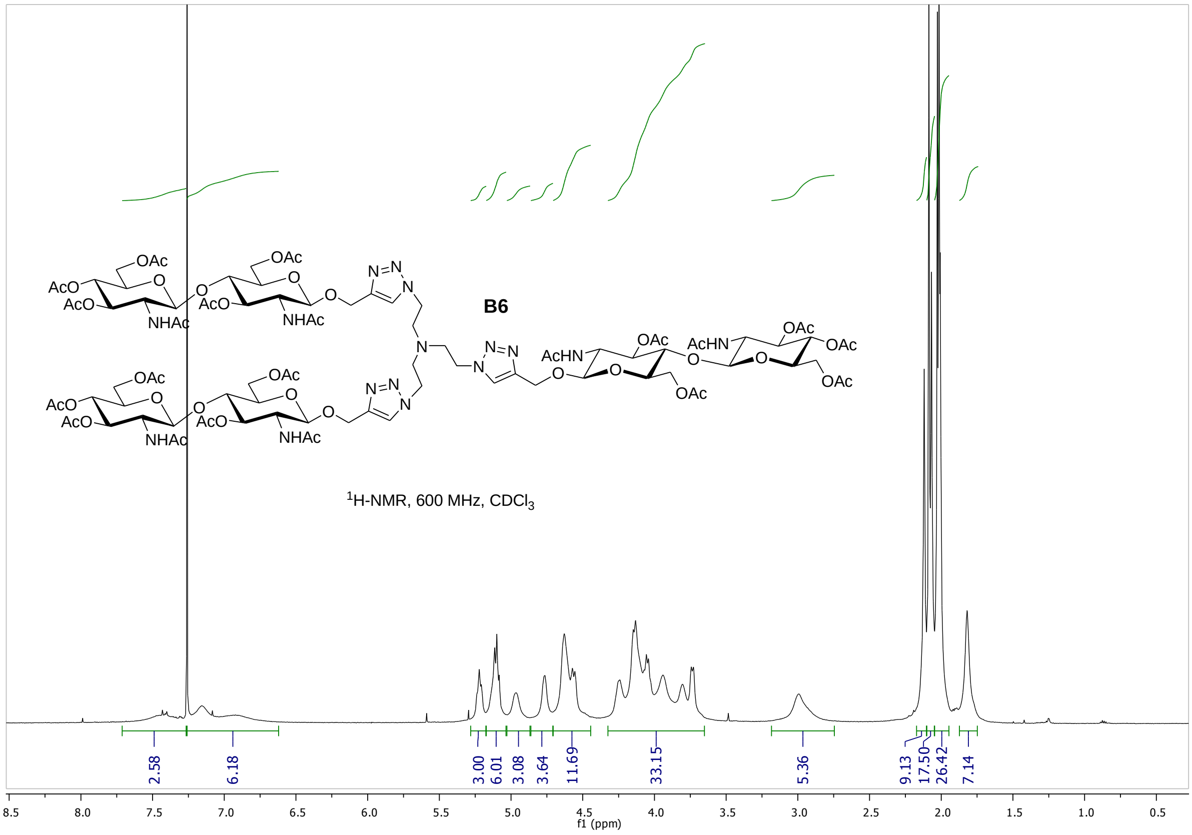
B6
¹H-NMR, 600 MHz, CDCl₃
f1 (ppm)

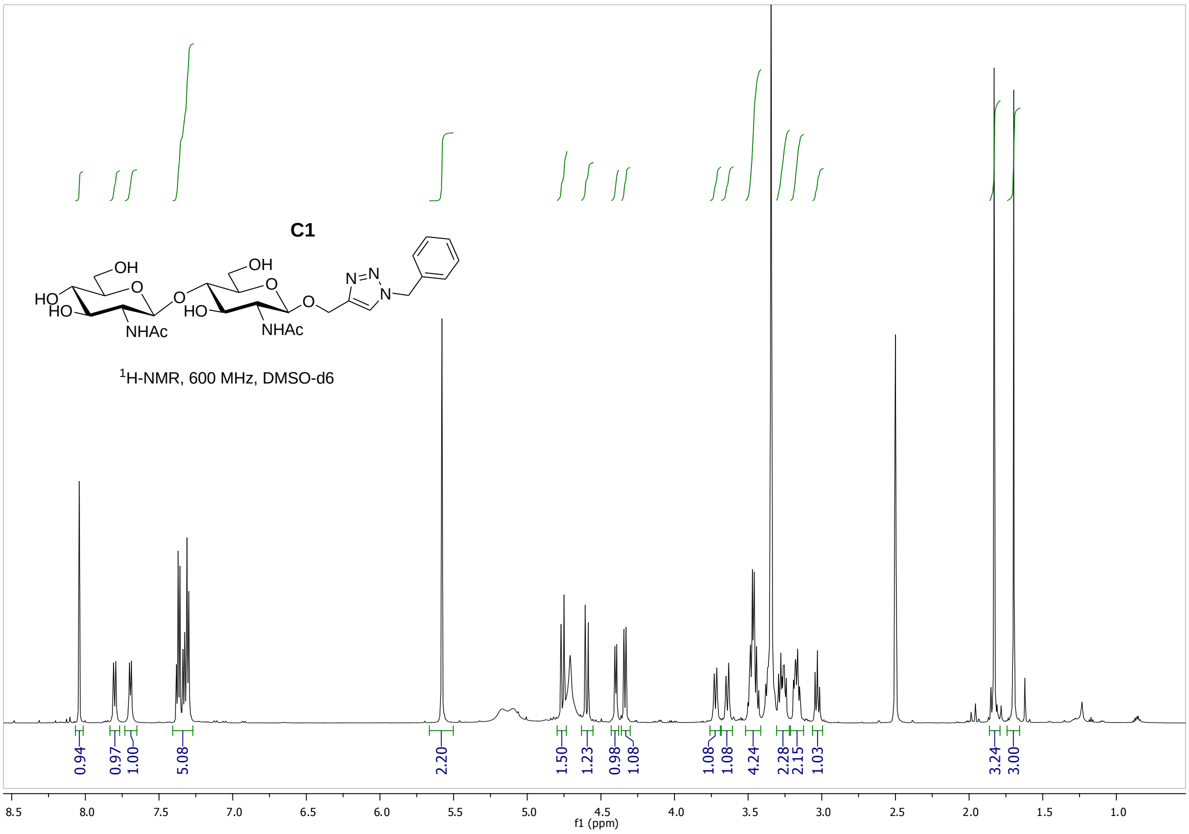
C1
OH
O
HO
HO
NHAc
O
OH
O
HO
NHAc
O
N=N
N
¹H-NMR, 600 MHz, DMSO-d6
0.94
0.97
1.00
5.08
2.20
1.50
1.23
0.98
1.08
1.08
1.08
4.24
2.28
2.15
1.03
3.24
3.00
8.5
8.0
7.5
7.0
6.5
6.0
5.5
5.0
4.5
4.0
3.5
3.0
2.5
2.0
1.5
1.0
f1 (ppm)

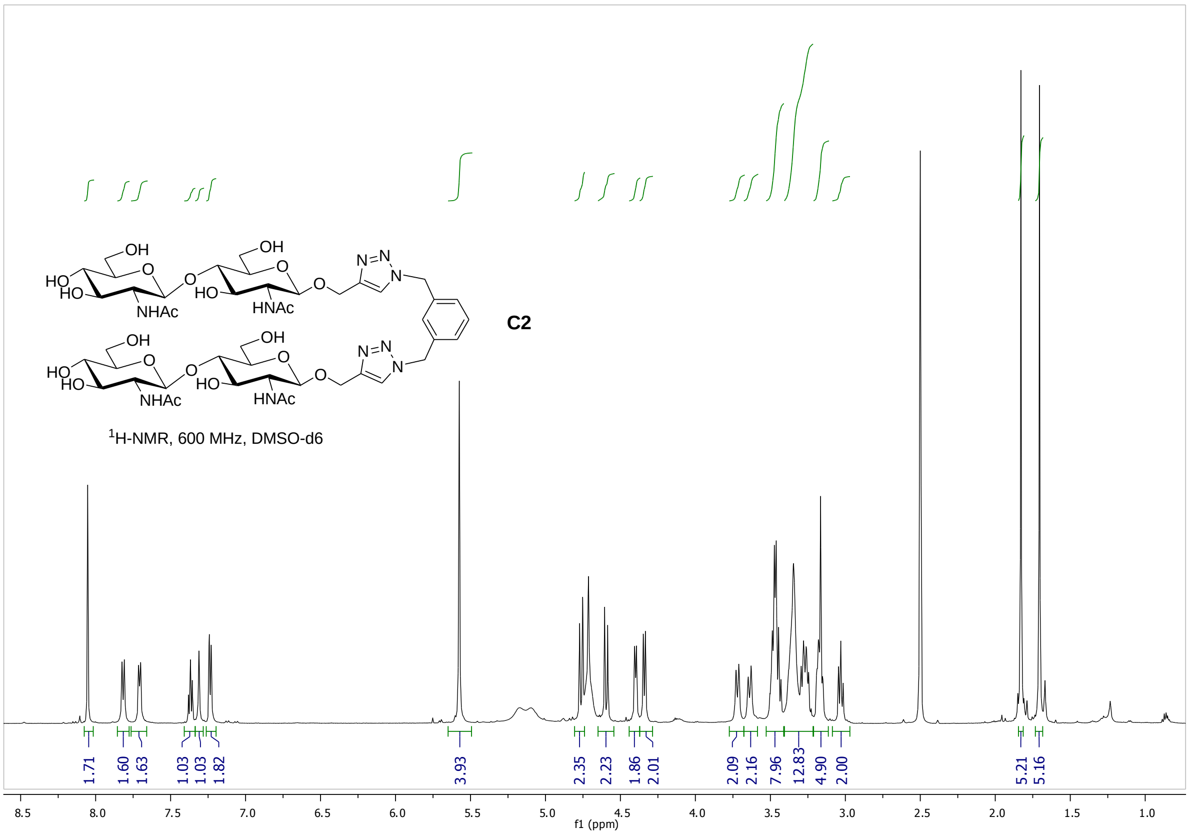
C2
HO
HO
NHAc
HNAc
¹H-NMR, 600 MHz, DMSO-d6
1.71
1.60
1.63
1.03
1.03
1.82
3.93
2.35
2.23
1.86
2.01
2.09
2.16
7.96
12.83
4.90
2.00
5.21
5.16
8.5
8.0
7.5
7.0
6.5
6.0
5.5
5.0
4.5
4.0
3.5
3.0
2.5
2.0
1.5
1.0
f1 (ppm)

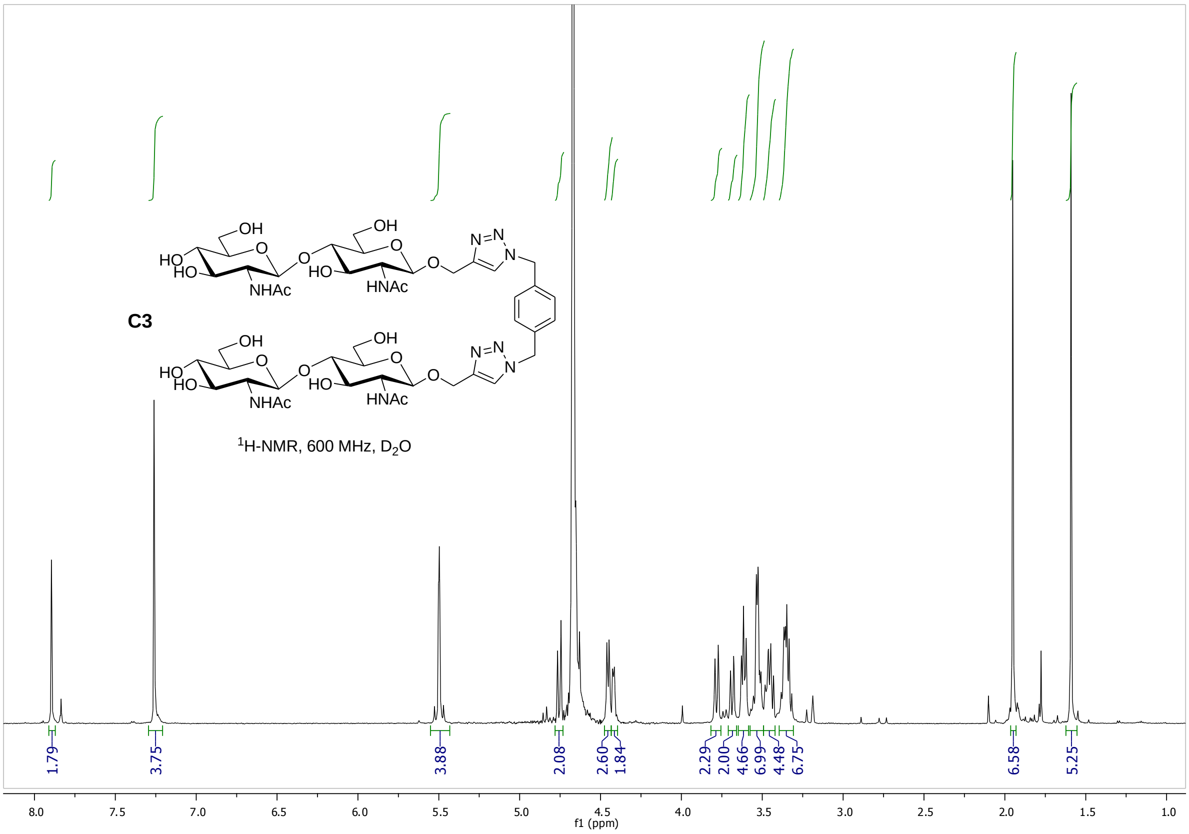
C3
¹H-NMR, 600 MHz, D₂O
1.79
3.75
3.88
2.08
2.60
1.84
2.29
2.00
4.66
6.99
4.48
6.75
6.58
5.25
f1 (ppm)

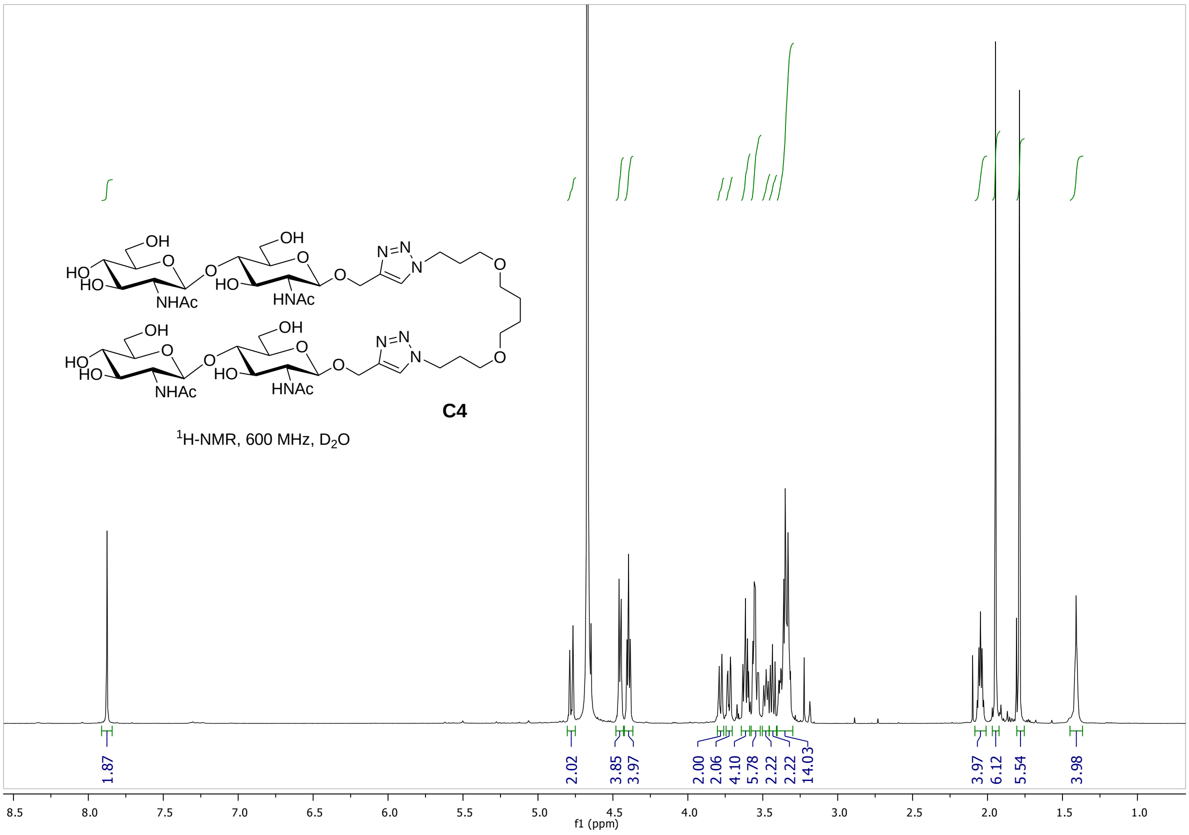
C4
¹H-NMR, 600 MHz, D₂O
1.87
2.02
3.85
3.97
2.00
2.06
4.10
5.78
2.22
2.22
14.03
3.97
6.12
5.54
3.98
f1 (ppm)

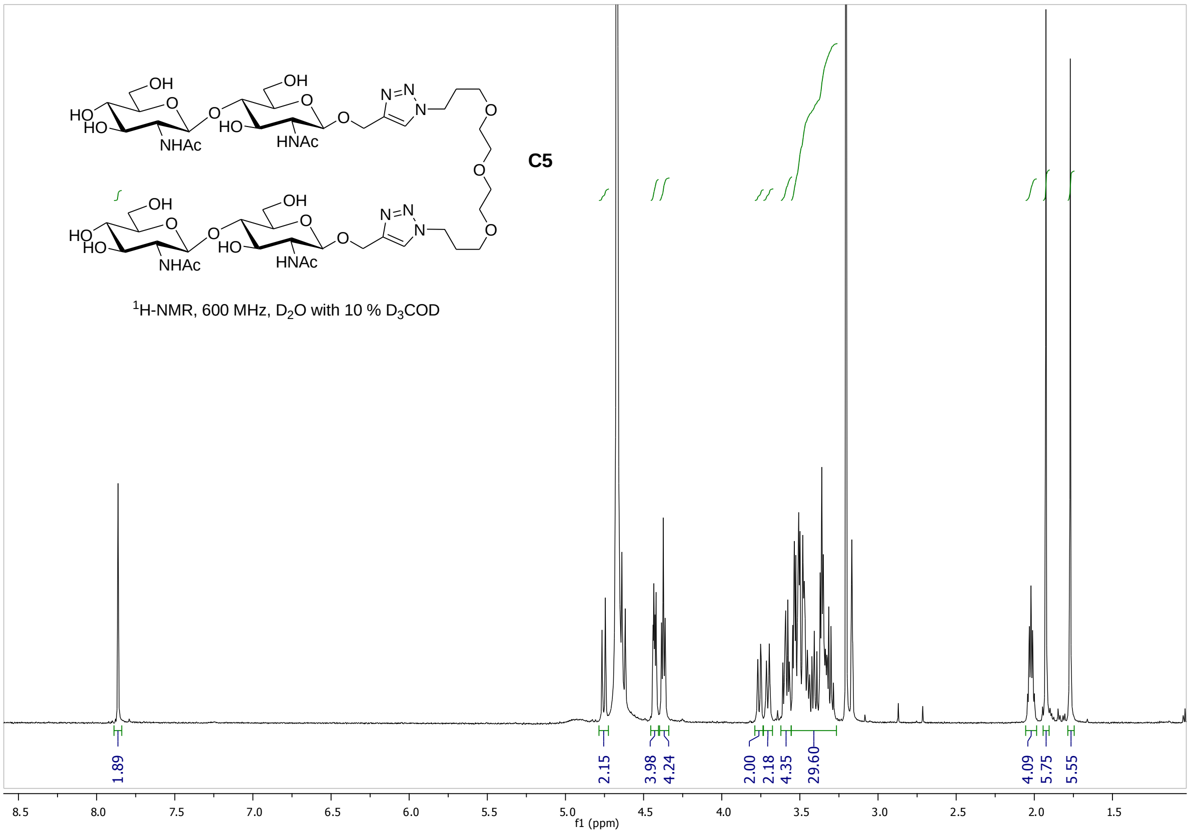
C5
1H-NMR, 600 MHz, D2O with 10 % D3COD
1.89
2.15
3.98
4.24
2.00
2.18
4.35
29.60
4.09
5.75
5.55
8.5 8.0 7.5 7.0 6.5 6.0 5.5 5.0 4.5 4.0 3.5 3.0 2.5 2.0 1.5
f1 (ppm)

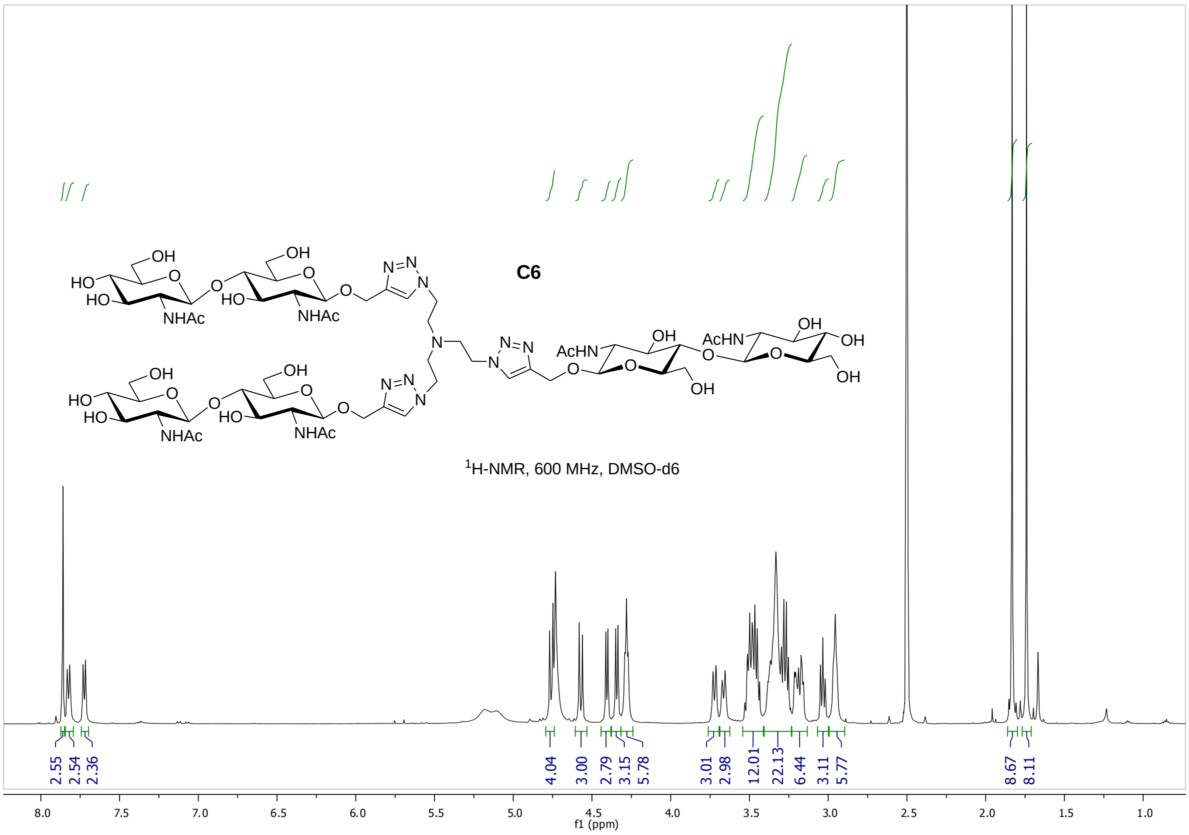
C6
1H-NMR, 600 MHz, DMSO-d6
2.55
2.54
2.36
4.04
3.00
2.79
3.15
5.78
3.01
2.98
12.01
22.13
6.44
3.11
5.77
8.67
8.11
8.0 7.5 7.0 6.5 6.0 5.5 5.0 4.5 4.0 3.5 3.0 2.5 2.0 1.5 1.0
f1 (ppm)
